# Anchor-based classification and type-C inhibitors for tyrosine kinases

**DOI:** 10.1038/srep10938

**Published:** 2015-06-16

**Authors:** Kai-Cheng Hsu, Tzu-Ying Sung, Chih-Ta Lin, Yi-Yuan Chiu, John T.-A. Hsu, Hui-Chen Hung, Chung-Ming Sun, Indrajeet Barve, Wen-Liang Chen, Wen-Chien Huang, Chin-Ting Huang, Chun-Hwa Chen, Jinn-Moon Yang

**Affiliations:** 1 Institute of Bioinformatics and Systems Biology, National Chiao Tung University, Hsinchu, Taiwan; 2 Department of Biological Science and Technology, National Chiao Tung University, Hsinchu, Taiwan; 3 Institute of Biotechnology and Pharmaceutical Research, National Health Research Institutes, Miaoli, Taiwan; 4 Department of Applied Chemistry, National Chiao Tung University, Hsinchu, Taiwan; 5 Department of Thoracic Surgery, Mackay Memorial Hospital, Taipei City, Taiwan

## Abstract

Tyrosine kinases regulate various biological processes and are drug targets for cancers. At present, the design of selective and anti-resistant inhibitors of kinases is an emergent task. Here, we inferred specific site-moiety maps containing two specific anchors to uncover a new binding pocket in the C-terminal hinge region by docking 4,680 kinase inhibitors into 51 protein kinases, and this finding provides an opportunity for the development of kinase inhibitors with high selectivity and anti-drug resistance. We present an anchor-based classification for tyrosine kinases and discover two type-C inhibitors, namely rosmarinic acid (RA) and EGCG, which occupy two and one specific anchors, respectively, by screening 118,759 natural compounds. Our profiling reveals that RA and EGCG selectively inhibit 3% (EGFR and SYK) and 14% of 64 kinases, respectively. According to the guide of our anchor model, we synthesized three RA derivatives with better potency. These type-C inhibitors are able to maintain activities for drug-resistant EGFR and decrease the invasion ability of breast cancer cells. Our results show that the type-C inhibitors occupying a new pocket are promising for cancer treatments due to their kinase selectivity and anti-drug resistance.

Protein kinases play a key role in regulating the phosphorylation of serine, threonine, or tyrosine residues of protein substrates^1^. Among the 518 protein kinases in the human genome, tyrosine kinases (TKs) is the largest subgroup and comprises the largest group of oncogenes^2^,^3^. TKs are considered attractive therapeutic targets against some diseases^3^,^4^ because they are often overexpressed in many tumor cells and play a critical role in many biological processes. Currently, there are two major issues in designing kinase inhibitors, namely low selectivity and drug resistance, because kinases share a structurally conserved ATP-binding site^5^ and the efficacy of kinase drugs is often limited by the development of drug resistance.

Current kinase inhibitors can be roughly divided into three types (I, II, and III). Most kinase drugs are type I inhibitors, which often target an active DFG-in conformation and directly compete with ATP, and thereby lack selectivity^6^. Type II inhibitors are often relatively selective because they bind to both ATP-binding site and an adjacent pocket by forming additional interactions with the αC-helix and the DFG motif 7. Type III inhibitors are non-ATP competitive inhibitors and often highly selective^8^. They interact with an allosteric region near the αC-helix and the DFG motif in the active state^6^. Although some TK inhibitors have entered clinical trials and more than 20 kinase drugs^9^,^10^ have been approved, most of them are promiscuous drugs that affect unexpected kinases^11^ and exhibit drug resistance. For example, gefitinib (Iressa) in lung cancer treatment targets 64 kinases (IC_50_ < 10 μM) among 356 tested kinases^7^, which results in diarrhea or liver damage. The above-mentioned studies suggest that the binding regions of kinase inhibitors limit the design of selective inhibitors.

Drug resistance has become a major problem in the treatment of cancers. Although TK drugs show promising efficacy in the clinic, most patients eventually relapse due to the development of drug resistance^12^,^13^. The development of drug resistance often results from mutations in the binding regions of the types I and II inhibitors. For example, the T790M mutation in epidermal growth factor receptor (EGFR) results in resistance to type I inhibitors^7^, such as gefitinib and erlotinib. Another example is the T315I mutation in ABL1. This mutation causes resistance to imatinib, which is a type II inhibitor^14^. Therefore, the discovery of new types of kinase inhibitors for overcoming drug resistance and kinase selectivity for diseases is a worthy goal.

Here, we quantified the binding-site properties of TKs and proposed a specific site-moiety map to explore the characteristics of the C-terminal hinge region, which is located in the C-terminal lobe and whose binding specificity is relatively unexplored. This region is located far from both the ATP-binding site and the binding regions of type I, II, and III inhibitors. Therefore, understanding the binding specificity of this region provides an opportunity to design selective and anti-drug resistant inhibitors. We have described site-moiety maps for elucidating protein-ligand binding mechanisms and discovering novel inhibitors for estrogen receptor, thymidine kinase, and shikimate kinase^15^,^16^,^17^,^18^.

In this paper, we develop specific site-moiety maps to understand the binding specificity and mechanisms of 51 protein kinases, including 45 TKs and 6 kinases belonging to the other kinase families. Based on these maps, we identified two specific anchors located in the C-terminal hinge region and presented a new classification of TKs for discovering new type-C inhibitors for TKs. Our experimental results show that type-C inhibitors are highly selective and can maintain their potency for drug-resistant EGFR because most interactions are provided by the C-terminal hinge region. In addition, type-C inhibitors block the invasion of breast cancer cells. We then synthesized several derivatives of type-C inhibitors to explore their binding mechanisms according to our anchor energy model and their moiety composition. These results reveal that type-C inhibitors are a good starting point for the design of a new type of kinase drugs due to their high selectivity and anti-drug resistance. We believe that our framework can be widely used to elucidate the binding mechanisms of the 518 human protein kinases in order to identify a novel inhibitor type.

## Results

### Overview of the discovery of binding specificity and a new type of inhibitors of tyrosine kinases

**Figure 1:**
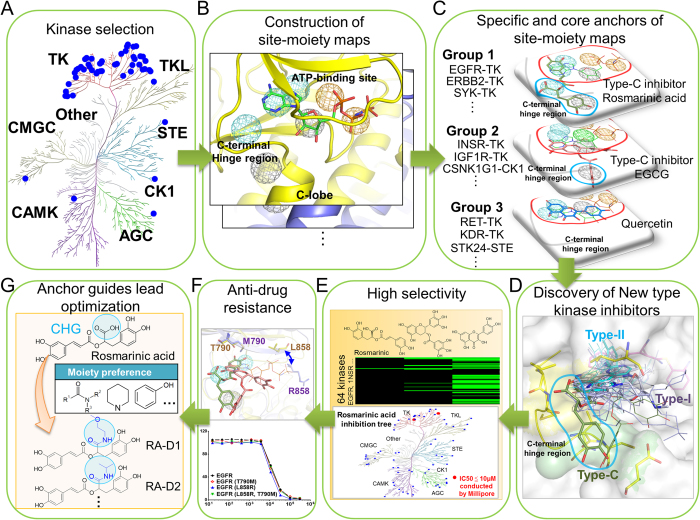
Overview of the quantification of binding specificity and discovery of type-C inhibitors for tyrosine kinases. (**A**) Selection of 51 protein kinases. In total, 45 tyrosine kinases and 6 protein kinases from six other kinase families were chosen. (**B**) Construction of site-moiety maps for these 51 kinases using 4,680 known kinase inhibitors. (**C**) Identification of specific anchors for type-C kinase inhibitors. The cyan and red circles indicate two specific and five conserved anchors in the C-terminal hinge region and the ATP-binding site, respectively. Tyrosine kinases are classified into three groups based on the presence of these two specific anchors. (**D**) Comparison of type-C, type-I, and type-II inhibitors. The type-C inhibitors (rosmarinic acid, green) seize the specific anchors in the C-terminal hinge region, whereas type-I and type-II inhibitors occupy the ATP-binding site. (**E**) Two type-C inhibitors (rosmarinic acid and EGCG) and quercetin were tested against 64 protein kinases. (**F**) Enzyme-based drug resistance assays of rosmarinic acid on EGFR. (**G**) Lead optimization of type-C inhibitors guided by our anchor model.

Figure 1 shows the major steps of constructing specific and core site-moiety maps for the binding specificity and a new type of inhibitors of TKs. Here, we focus on exploring the characteristics of the C-terminal hinge region and discovering new type-C inhibitors, which bind to this pocket and can overcome the drug-resistant mutations acquired by type I and II inhibitors. We first collected 45 TKs with available X-ray structures. To compare with other protein kinases, we selected 6 protein kinases from the other kinase families^19^ (Fig. 1A and Table S1 in the supporting materials): AGC, CAMK, CK1, CMGC, STE, and TKL. We then constructed site-moiety maps for these 51 protein kinases by docking 4,680 known kinase inhibitors collected from the BindingDB database^20^ into each kinase using GEMDOCK^21^,^22^, an in-house docking tool. GEMDOCK has been successfully applied to the discovery of novel inhibitors and binding mechanisms for some target proteins^23^ and has yielded comparable molecular docking and screening performance to other docking tools, such as FlexX and GOLD^21^. The top 2,000 inhibitors of each kinase ranked by docking energy were selected to establish its site-moiety map, which represents the characteristics of the C-terminal hinge region and the ATP-binding site of the kinase (Fig. 1B). We developed a method to align these constructed 51 site-moiety maps for identifying the specific and core site-moiety maps of these selected kinases (Fig. 1C). The alignment of these site-moiety maps include two specific anchors, which are possessed by several protein kinases (e.g., EGFR, ERBB2, and SYK), and five core anchors, which are conserved in more than 90% protein kinases (Fig. 1C). The specific and core anchors can represent the selectivity-determining and common characteristics of the C-terminal hinge region and the ATP-binding site, respectively. Furthermore, we clustered these 51 kinases into three groups according to these core and specific anchors (Fig. 1C).

We then used the site-moiety maps to discover type-C kinase inhibitors (Fig. 1D) by screening 118,759 natural compounds collected from the ZINC compound database^24^. The type-C inhibitors should be more selective (Fig. 1E) and less susceptible to drug resistance than type I and II inhibitors because the C-terminal hinge region is farther from the ATP-binding site (Fig. 1F). We identified two potential type-C inhibitors, namely rosmarinic acid and EGCG, which match both specific anchors and one specific anchor, respectively. In addition, quercetin, which lies in the ATP-binding site and matches only core anchors was selected as a potential broad-spectrum inhibitor. These three compounds were tested on 64 proteins selected from all kinase families to verify their kinase selectivity (Fig. 1E). Based on our anchor model, we synthesized four derivatives of rosmarinic acid to understand the binding mechanisms of the specific anchors (Fig. 1G). Finally, two type-C inhibitors and their derivatives can maintain their potency for drug-resistant EGFR and decrease the invasion of breast cancer cells.

### Core anchors in the ATP-binding site

The 51 aligned site-moiety maps consist of two specific and five core anchors in the C-terminal hinge region and the ATP-binding site, respectively (Figs. S1 and S2 in the supporting materials). The five core anchors, which represent the common characteristics of the protein kinases, are named the pyrimidine ring of adenine (PA), the imidazole of adenine (IA), ribose (RB), α-phosphate (AP), and β-phosphate (BP) anchors, according to their locations (Figs. S2A and S2B in the supporting materials). We used the residue numbering of EGFR as the reference for describing the anchors. The PA and IA anchors, which occupy the adenine position of ANP and ATP, have similar moiety preferences. Their interacting moieties often form hydrogen-bonding and van der Waals interactions with anchor residues (such as L718, V726, Q791, M793, and L844). Many known kinase inhibitors and drugs (e.g., gefitinib and erlotinib) have been designed with polar ring moieties to mimic the adenine in these two anchors. The RB anchor, which is situated in the ribose-binding region, is mainly formed by van der Waals interactions, such as ring moieties. Two remaining anchors, namely AP and BP, are located adjacent to the α-phosphate and β-phosphate moieties of ATP, respectively. These results show that our site-moiety maps can identify the conserved interacting residues and preferred moieties to reflect the ATP binding mechanisms and protein kinase functions. The details of the core site-moiety maps are described in the supporting materials.

### Specific anchors in the C-terminal hinge

**Figure 2:**
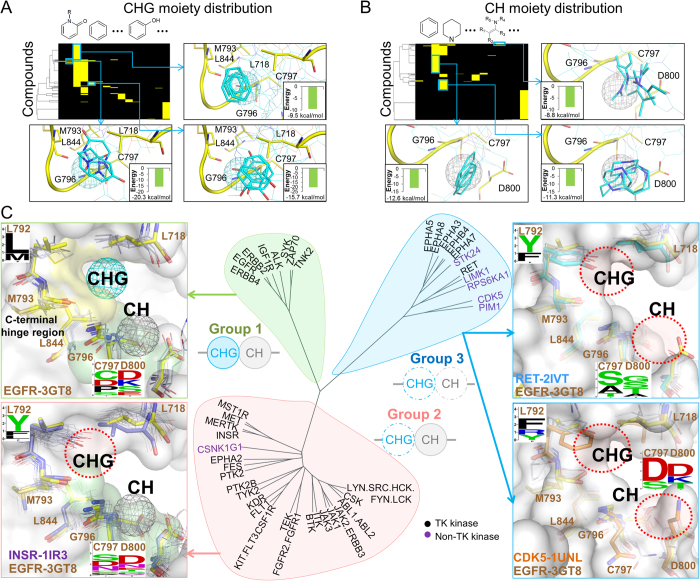
Anchor-based classification of tyrosine kinases. Interacting moiety profiles of (**A**) CHG and (**B**) CH anchors. An entry is colored yellow if the compound has a moiety that interacts with the anchor residues; otherwise, the entry is colored black. The frequently interacting functional groups and their average binding energy are shown near the profile. The moieties with a low binding energy can be used for the lead optimization process. (**C**) Classification tree and anchor residue patterns of three groups. The tree was constructed by considering the anchor presence and the anchor residue identities. Group 1 kinases contain CH and CHG anchors. The structures of EGFR and the other Group 1 kinases are shown as yellow stick and white line representations, respectively. Group 2 kinases lack the CHG anchor because of the long and rigid residue types (residue F/Y) at the 792 position near the CHG anchor. In Group 3 kinases, the short side chains of the residues at the positions 793 and 796 result in elimination of the CH and CHG anchors.

The first specific anchor, which was denoted CH for “near the C-terminal hinge” (Figs. S2A and S2C in the supporting materials), consists of the interacting residues G796, C797, and D800, which form a binding pocket based on the residue numbering of EGFR (PDB code 3GT8). The second specific anchor is the CHG anchor, which is located between the C-terminal hinge and G-loop, and consists of five interacting residues: L718, M793, G796, C797, and L844. The sequence profiles of the CH and CHG anchor residues show that the residues at positions 797 and 800 are diverse in the TK family despite their sequence similarities (Fig. S3A in the supporting materials). Conversely, the protein kinases in other families exhibit relatively high conservation at these two positions than TKs. These two specific anchors in the C-terminal hinge region provide a new clue to reveal the binding specificity and selective inhibitors of TKs. In addition, these moieties can form stable interactions with the anchor residues and can be used for guiding lead optimization (Fig. 2A,B).

### Anchor-based classification of tyrosine kinases

Based on the presence of these two specific anchors (CH and CHG), we clustered the TKs into three groups (Fig. 2C and Table S1 in the supporting materials). Group 1 consists of 8 TKs that have both CH and CHG anchors: ALK, EGFR, ERBB2, ERBB4, IGF1R, SYK, TNK2, and ZAP70. In this group, most of the CHG anchor residues (i.e., at 718, 793, 796, and 844 positions) have similar physicochemical properties. Although the kinases in Group 1 have different residue types at positions 797 and 800, at least one anchor residue (e.g., residues C/D and D/E/K at positions 797 and 800, respectively) with a long side chain exhibited a similar orientation to form the pocket of the CH anchor (Fig. S3B in the supporting materials). For example, the residue types of EGFR and SYK are D and K at position 800, respectively, and their orientations are similar to form the binding pocket (Fig. 2C).

Among the 51 protein kinases tested, 31 kinases only contain the CH anchor and comprise Group 2, such as CSNK1G1, INSR, KIT, MET, and SRC. Group 2 kinases have at least one long side-chain residue at the positions 797 or 800 to yield the CH anchor, similarly to the Group 1 kinases. However, the Group 2 kinases lack the CHG anchor because the aromatic residues (i.e., F/Y) at position 792 limit the formation of the CHG anchor (Figs. 2C and S3B in the supporting materials). The side chains of the aromatic residues are bulky and point toward the pocket center of the CHG anchor, preventing the binding of compounds. In comparison to the Group 1 kinases, the side chain of residue 792 (L or M) is flexible and can accompany with residues 793 and 844 to yield the CHG pocket. In addition, several kinases are classified as Group 2 instead of Group 1 because the Group 2 residue located at position 718 points toward the C-terminal hinge region and eliminates the CHG anchor pocket. The additional analysis of anchor-based classification is described in the supporting materials.

The anchor-based Group 3 contains 11 kinases containing only core anchors. These 11 kinases cover 6 protein families, which suggests that the CHG and CH anchors may be specific for TKs. Similar to the Group 2 kinases, the Group 3 kinases have bulky residues (i.e., F/Y/R) at position 792 and therefore lack the CHG pocket. For elimination of the CH anchor in this group, the side-chain orientation of the residue at the position 800 plays a key role. More details and examples (Fig. S4 in the supporting materials) of the anchor-based are described in the supporting materials.

### Identification of type-C inhibitors

We identified type-C inhibitors that match the specific anchors by performing a large-scale virtual screening of natural compounds against 9 selected representative protein kinases (Fig. S1 in the supporting materials). These 9 kinases were selected from three anchor-based groups: EGFR, SYK, and IGF1R in Group 1; INSR, CSNK1G, and ABL1 in Group 2; RET, STK24, and CDK5 in Group 3. Natural compounds are promising sources for developing new drugs because more than 80% of drugs were developed based on the structures of natural compounds^25^. For each representative protein kinase, we docked 118,759 natural compounds collected from the ZINC compound database^24^ into the binding site using the in-house tool GEMDOCK running on a PC cluster with 100 CPU cores. After screening these natural compounds on 9 selected kinases, the docked compounds were divided into three groups: matching both of the CH and CHG anchors (Group 1), only matching the CH anchor (Group 2), and matching only core anchors (Group 3). For each group, the compounds were ranked by the total site-moiety map scores. The compounds occupying both specific and core anchors were considered potential type-C inhibitors.

The total site-moiety map score of compound *x* was defined as 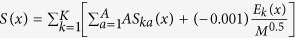, where *AS*_*ka*_(*x*) is the anchor score of compound *x* in anchor *a* of kinase *k*, *A* is the anchor number of kinase *k, K* is the number of the selected representative kinases, *E*_*k*_(*x*) is the docked energy of compound *x* in kinase *k* generated by GEMDOCK, and *M* is the atom number of compound *x. AS*_*ka*_(*x*) is set to 1 if compound *x* matches anchor *a* of kinase *k*; otherwise it is set to 0. For example, the value of *A* for EGFR equals 7, and the *K* value equals 3 for the selected kinases (i.e., EFGR, SYK, and IGF1R) of Group 1. The term *M*^0.5^ is used to reduce the deleterious effect of selecting high-molecular-weight compounds.

**Figure 3:**
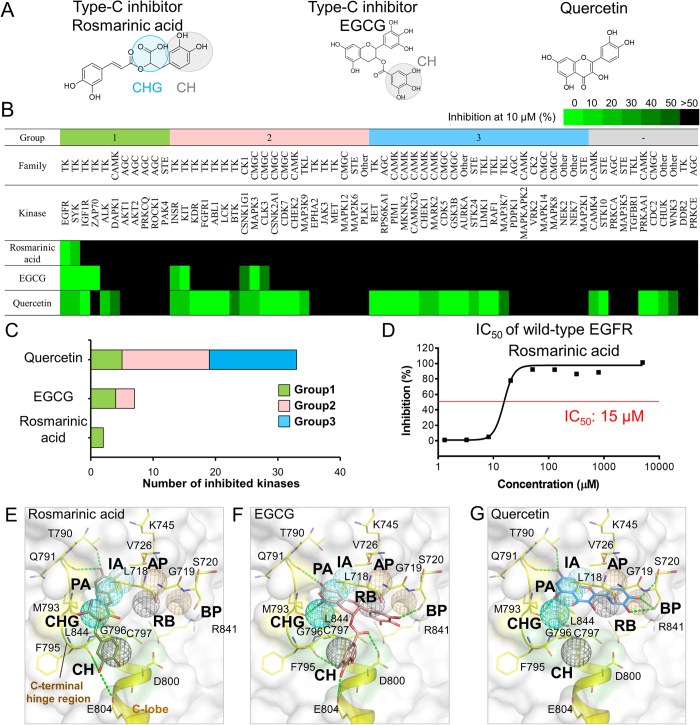
Selectivity of type-C inhibitors and quercetin. (**A**) Structures of the compounds. Rosmarinic acid and EGCG are type-C inhibitors, which match two (CH and CHG) specific anchors and one (CH) specific anchor, respectively. In comparison, quercetin matches none of the specific anchors. (**B**) Results of compound profiling. The percentage of the remaining kinase activity is indicated from green (0%) to black (>50%). A compound is considered a potential inhibitor against kinases if the percentage of the remaining kinase activity is ≤50% at a compound concentration of 10 μM. (**C**) The target number of three compounds. Rosmarinic acid, EGCG, and quercetin inhibit 2 (3%), 9 (14%), and 39 (61%) of the 64 protein kinases, respectively. (**D**) IC_50_ value of rosmarinic acid for wild-type EGFR. Docked poses of the compounds (**E**) rosmarinic acid, (**F**) EGCG, and (**G**) quercetin in EGFR. Hydrogen-bonding interactions are represented as green dashes.

To identify potential type-C inhibitors and verify the specific anchors, we selected several natural compounds from the top-ranked 2,000 compounds for kinase profiling (Table S2 in the supporting materials). Based on the total site-moiety map score, drug-like properties, domain knowledge, and availabilities, we selected two type-C compounds, namely rosmarinic acid (Group 1) and EGCG (Group 2) for kinase profiling (Fig. 3). We also selected a well-known natural compound, quercetin (Group 3), for comparing type-C compounds (Fig. 3A). Rosmarinic acid is commonly found in plants of the family *Boraginaceae* and has antiviral, anti-inflammatory, and various other biological effects^26^. EGCG is a major component of catechin derivatives of green tea, and quercetin is a flavonoid that is widely found in many plants. These natural compounds have been documented as exerting several chemopreventive and anticancer effects^27^,^28^. However, the selective mechanisms of these three natural compounds on protein kinases remain unclear, particularly for rosmarinic acid.

### Kinase profiling for type-C inhibitors

Here, we used kinase profiling to evaluate the specific anchors and to elucidate the selectivity of the two potential type-C inhibitors and quercetin against 64 protein kinases selected from different kinase families. The kinase profiles were obtained using the KinaseProfiler Service offered by Millipore (Table S3 in the supporting materials). Rosmarinic acid, EGCG, and quercetin were tested at concentrations of 10 μM, and the percentage of the remaining kinase activity (RKA) was used as a metric for the inhibitory ability. Here, a compound was considered to be an inhibitor against a kinase if the percentage of RKA ≤50%, which denotes that its IC_50_ value is often less than 10 μM for the tested kinase. The experimental results of the profiling indicate that rosmarinic acid (Group 1) is the most selective inhibitor (Fig. 3B,C) and inhibits two TKs, EGFR (RKA = 9%) and SYK in Group 1. EGCG (Group 2) matches one specific anchor and inhibits nine kinases, including EGFR (RKA ≤ 0%), SYK, IGF1R and ZAP70 (RKA = 4%) in Group 1, INSR, KIT (RKA = 4%) and CSNK1G1 in Group 2, and MAPK3 and CLK3 in the CMGC family (Fig. 3B and Table S3 in the supporting materials). Of these nine protein kinases, the majority (67%) belong to the TK family and are included in Groups 1 and 2. The kinases of these two groups consistently contain the CH anchor and are thereby able to be inhibited by EGCG. In addition, quercetin (Group 3) is the most broad-spectrum inhibitor, inhibiting 39 (61%) of the 64 protein kinases distributed across all of the protein kinase families and the three groups (Figs. 3B,C). We further conducted an enzyme-based assay for rosmarinic acid, and the IC_50_ for EGFR is 15 μM (Fig. 3D). These experimental results show that the specific anchors can reveal the kinase selectivity and the type-C inhibitors are highly selective and potentially provide an opportunity for developing a new type of kinase drugs.

### Binding modes of type-C inhibitors

The type-C inhibitor of Group 1, rosmarinic acid, matches two specific anchors, CH and CHG, in the C-terminal hinge region and is the most selective inhibitor tested in this study. The docked pose of rosmarinic acid shows that one of its catechol groups occupies the pocket of the specific anchor CH, and interacts with the anchor residues G796 and C797 (Fig. 3E). This catechol also forms two hydrogen bonds with residues E804 and F795 in the C-terminal hinge region. The 2-hydroxypropanoic acid of rosmarinic acid forms van der Waals interactions with residues L718, M793, and G796 of the CHG anchor. These specific interactions account for the high selectivity of the type-C inhibitor, rosmarinic acid, for Group 1 kinases. In comparison, rosmarinic acid shows no inhibition against the kinases INSR (Group 2) and RET (Group 3) because they lose the CHG anchor (Figs. S5A and S5B in the supporting materials).

EGCG is considered a type-C inhibitor of Group 2 and only matches one specific anchor (CH) (Fig. 3F). The gallate moiety of EGCG generates van der Waals interactions with the specific anchor residues G796, C797, and D800. The hydroxyl groups of the gallate yield hydrogen bonds with residues F795 and E804. Moreover, the ketone moiety near the gallate forms a hydrogen-bonding interaction with residue D800. These specific interactions in the CH anchor may contribute to the selectivity of EGCG. For example, the RET kinase belongs to the TK family and is poorly inhibited by EGCG because it lacks the CH anchor (Fig. S5C and S5D in the supporting materials). Quercetin shows the broadest protein kinase inhibitory activity in the kinase profiling assay because it matches three core anchors (PA, IA, and RB) without any specific anchors (Fig. 3G). The details of binding mode of quercetin are described in the supporting materials.

### Synthesis of type-C inhibitors

To the best of our knowledge, our model is the first to show that the CH and CHG anchors in the C-terminal hinge region can be used to design highly selective kinase inhibitors. According to the analysis of 17 kinase-drug complex structures, none of the kinase drugs and inhibitors target both CH and CHG anchors (Fig. 1D). Based on the enzymatic bioassay results, rosmarinic acid has high binding specificity and provides a great potential value for the development of type-C kinase inhibitors for TKs.

**Figure 4:**
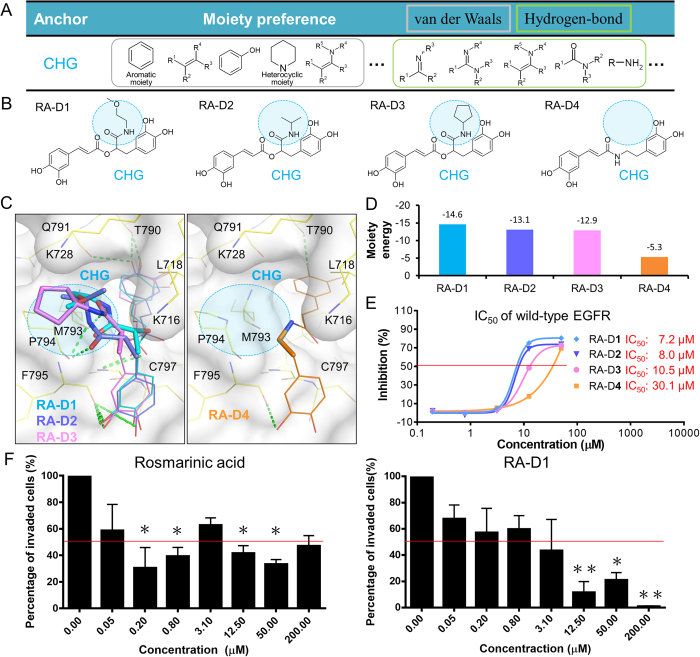
Specific anchor guides the synthesis of rosmarinic acid derivatives. (**A**) Moiety preferences of CHG anchor. (**B**) Four rosmarinic acid derivatives, namely RA-D1, RA-D2, RA-D3, and RA-D4, were synthesized based on the moiety composition of the CHG anchor. (**C**)The moieties located at the CHG anchor are represented as sticks, and the other moieties are represented as lines. (**D**) Interaction energy between the substituted moieties and residues of the CHG anchor. (**E**) Dose-response curves and IC_50_ values of the derivatives. The first three derivatives have large substituted moieties that form van der Waals interactions with residues of the CHG anchor and consistently present better activity compared with rosmarinic acid. In comparison, RA-D4 lacks a moiety in the CHG anchor and shows poor inhibitory activity. (**F**) Effect of type-C inhibitors on invasion of MDA-MB-231 breast cancer cells. The invasion activity was tested using Corning BioCoat Matrigel Invasion Chambers. *, *p* < 0.05; **, *p* < 0.01, compared with the control group.

Based on the specific anchors and the binding mode of rosmarinic acid, we synthesized its four derivatives (i.e., RA-D1, RA-D2, RA-D3, and RA-D4) to understand the binding mechanisms on the CHG anchor (Fig. 4). Because the binding pocket of the CHG anchor prefers to form van der Waals interactions with large moieties, we substituted the carboxylic acid of rosmarinic acid with N-(1-methoxypropan-2-yl) formamide (RA-D1), N-isopropylformamide (RA-D2), and N-cyclopentylformamide (RA-D3) (Fig. 4A,B). In addition, a compound (RA-D4) without the carboxylic acid was synthesized for the comparison with the first three derivatives. The docked poses show that the substituted moieties of compounds RA-D1, RA-D2, and RA-D3 consistently provide additional van der Waals contacts with residues L718, M793, and P794 (Fig. 4C) compared with the carboxylic acid of rosmarinic acid. The interaction energies between the substituted moieties of compounds RA-D1, RA-D2, and RA-D3 and the residues of the CHG anchor are –14.6, –13.1, and –12.9 kcal/mol (Fig. 4D), respectively, as calculated by the scoring function of GEMDOCK^21^,^22^. Therefore, the three derivatives have better activities than rosmarinic acid. RA-D1, RA-D2, and RA-D3 inhibit EGFR with IC_50_ values of 7.2, 8, and 10.5 μM (Fig. 4E), respectively, whereas the IC_50_ value of rosmarinic acid is 15 μM. In comparison, the interaction energy of RA-D4 is higher due to the lack of the carboxylic acid and results in a poor inhibitory activity (IC_50_ value: 30.1 μM). These observations reveal that the moieties forming van der Waals interactions in the CHG pocket are essential for the activity of type-C inhibitors and that the moiety energies in the anchors can be used for lead optimization.

### Type-C inhibitors suppress the invasion of breast cancer cell

The invasion of cancer cells is one of the crucial steps for metastasis. Various growth factor receptors, such as EGFR, VEGFR, and MET, are associated with the invasion process of cancer cells^29^,^30^,^31^. Therefore, inhibitions of these proteins are considered an effective strategy for preventing cancer cell invasion. In breast cancer, EGFR is overexpressed in 20 ~ 50% of patients and plays a key role in promoting invasion^32^. Here, we tested two type-C inhibitors (i.e., rosmarinic acid and RA-D1) through invasion assays using the MDA-MB-231 breast cancer cell line.

We first determined the non-cytotoxic concentrations of the type-C inhibitors using the CellTiter-Glo Luminescent Cell Viability Assay before performing the invasion assay because the invasion assay needs to be performed at non-cytotoxic concentrations. The CellTiter-Glo Luminescent Cell Viability Assay was applied to evaluate cell viability after treatment with rosmarinic acid and RA-D1. The experimental results show that the compounds have no cytotoxic effects against the cells in the concentration range from 0.05 to 50 μM (Fig. S6 in the supporting materials). When the concentration reached 200 μM, the cell viability decreased to approximately 50%. The inhibition of MDA-MB-231 breast cancer cell invasion was then determined when the two compounds were tested at doses between 0.05 and 200 μM using Corning BioCoat Matrigel Invasion Chambers. Rosmarinic acid and RA-D1 induced a dose-dependent decrease in cell invasion through the matrigel (Fig. 4F). The percentages of invaded cells were reduced by 42% and 44% after treatment with 12.5 μM rosmarinic acid and 3.1 μM RA-D1, respectively. The experimental results show that these two compounds suppress the invasion of breast cancer cells and potentially prevent cancer metastasis.

### Type-C inhibitors maintain their potency for drug-resistant EGFR

In the clinic, the efficacy of TK drugs (e.g., gefitinib and erlotinib) is often limited during the treatment due to the presence of drug-resistance mutations, such as T790M and L858R in EGFR^33^,^34^, Y253H, E255V, and T315I in ABL1^35^, T474I in BTK^36^, and V617F in JAK2^37^. These mutations often occur in the ATP-binding site and thereby cause steric interference or hinder optimal hydrogen-bonding interactions with the hinge, which decreases the affinity for TK drugs^38^,^39^. Among these mutations, T790M, the gatekeeper mutation in EGFR, was observed in 50% of clinically drug-resistant patients^12^,^40^. These mutations are less likely to affect the binding of type-C inhibitors to TKs because most interactions between EGFR and type-C inhibitors (RA and EGCG) are contributed from residues of the C-terminal hinge region.

**Figure 5:**
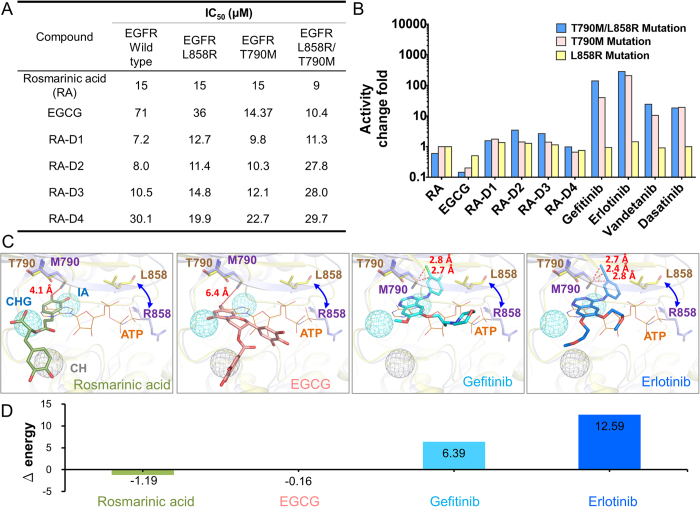
Type-C inhibitors against drug-resistant EGFR. (**A**) IC_50_ values of type-C inhibitors against wild-type, T790M, L858R, and T790M/L858R mutant EGFR. (**B**) Fold changes in activity of rosmarinic acid, EGCG, gefitinib, erlotinib, vandetanib, and dasatinib in the presence of the drug-resistant mutations. (**C**) Binding modes of type-C inhibitors and kinase drugs in the T790M/L858R mutant EGFR. The kinase-drug complex structures and the mutant EGFR structure (PDB 3W2P) are superimposed on the structure of wild-type EGFR, including EGFR-gefitinib (PDB 2IVT) and EGFR-erlotinib (PDB 1M17) complex structures. (**D**) Energy difference of the compounds between the wild-type (T790) and mutant (M790) residues calculated by GEMDOCK. Type-C inhibitors maintain similar interaction energy for the mutant residue, whereas gefitinib and erlotinib lose interactions when the mutation occurs.

We tested the type-C inhibitors against the wild-type and drug-resistant EGFR proteins. The enzyme-based assays showed that rosmarinic acid inhibits wild-type, T790M, L858R, and T790M/L858R EGFR with IC_50_ values of 15, 15, 15, and 9 μM, respectively (Fig. 5A). Similarly, the rosmarinic acid derivatives and EGCG maintain their potency for these EGFR mutations. Conversely, the T790M/L858R mutation causes approximately 140, 284, 34, and 18-fold reduction in the EGFR inhibition for gefitinib, erlotinib, vandetanib, and dasatinib, respectively (Fig. 5B)^7^.

The T790M/L858R mutation leads to a conformational rearrangement of the N-lobe and maintains EGFR in an active-like conformation^38^, which may disrupt the optimal interactions required for inhibitor binding. Because most kinase drugs rely on interactions with the gatekeeper residue or other residues of the ATP-binding site, they may be susceptible to the mutations in the regions (Fig. 5C). For example, gefitinib and erlotinib are near (2.7 Å and 2.4 Å, respectively) the mutant residue M790 (Fig. 5C). The differences in the energy of gefitinib and erlotinib between the wild-type and mutant EGFR proteins are 6.3 and 12.6 kcal/mol (Fig. 5D), respectively, as obtained by GEMDOCK^21^,^22^. This may change the binding conformations of the drugs and thus affect their effectiveness. In contrast, the type-C inhibitors rosmarinic acid and EGCG are far from the methionine ( >4 Å) and have similar energies with the wild-type and mutant EGFR proteins. Furthermore, the main interactions between the type-C inhibitors and EGFR are yielded by the residues in the C-terminal hinge region. These experimental results suggest that type-C inhibitors can maintain their potency for drug-resistance mutations and are a good starting point for the design of anti-resistant drugs.

## Discussion

Here, we present the use of specific site-moiety maps for discovering the binding specificity and a new type of inhibitors of TKs, which has two advantages. First, specific anchors can be used to represent the binding specificity of the C-terminal hinge region. Our previous studies showed that anchors are often essential for molecular recognition processes^15^,^16^. In addition, the moiety preferences of anchors provide clues for the lead optimization process. RA-D1, RA-D2, and RA-D3 inhibit EGFR with better potency than rosmarinic acid because they have lower binding energies with the CHG anchor residues (Fig. 4). In contrast, RA-D4 has poor potency due to the elimination of the carboxyl group in the CHG anchor.

Second, we used an anchor-based alignment of these 51 protein kinases to discover the binding specificity of these kinases instead of sequence or structure alignments. Our model is particularly useful for distinguishing specific characteristics from kinases with similar sequences in order to design selective inhibitors. For example, EGFR and SYK belong to Group 1 but do not share sequence similarity in the C-terminal hinge region (Fig. S3B in the supporting materials). Based on their anchor-based classifications, EGFR and SYK share similar physicochemical properties and are simultaneously inhibited by rosmarinic acid. Another example is KIT and CSNK1G1, which belong to the TK and CK1 families according to their sequence similarity, respectively, but these proteins have the CH anchor and share the same inhibitors, EGCG and rosmarinic acid. Our anchor model is more likely to reflect the physicochemical properties than the sequence-based classification.

The design of a new type of kinase inhibitors is an essential therapeutic strategy. Many kinase inhibitors have failed in clinical trials and have resulted in treatment failure because of low selectivity and the emergence of drug resistance. Among the natural compounds tested here, rosmarinic acid is a highly selective inhibitor for EGFR and SYK. EGFR is a cell-surface receptor and has been considered an attractive therapeutic target in cancer therapy^12^,^33^. SYK is a key modulator of immune signaling and has been shown to be highly expressed in rheumatoid arthritis^41^. Therefore, we propose that rosmarinic acid is a good starting point for developing a new type of inhibitors for the treatment of non-small-cell lung cancer with drug-resistant EGFR and rheumatoid arthritis.

EGCG may be a useful multiple-kinase agent for the treatment of complex diseases, such as cancers. For example, EGCG was found to inhibit nine protein kinases in this study, three (EGFR, KIT, and IGF1R) of which are overexpressed or mutated in lung cancer cells^12^,^42^,^43^. Hence, EGCG can be effectively employed in lung cancer because it simultaneously inhibits these three protein kinases. In addition, EGCG occupying the C-terminal hinge region can maintain its effectiveness for the inhibition of EGFR with the T790M, L858R, and T790M/L858R mutations and has a low probability of encountering drug resistance because the probability of resistance-conferring mutations simultaneously arising in the three proteins is exponentially low. This point is particularly important for cancer therapy due to the high mutation rate of cancer cells.

The lack of selectivity and the emergence of drug resistance are the major obstacles in the development of protein kinase inhibitors. In this study, we developed specific site-moiety maps to reveal the binding specificity of TKs and identified two specific anchors in the C-terminal hinge region. Through the specific anchors, we discovered and synthesized a new type (type-C) of kinase inhibitors. The type-C inhibitors have high selectivity and overcome drug resistance. We believe that specific site-moiety maps can be applied to elucidate binding mechanisms and to discover selective inhibitors for all 518 human protein kinases. Our findings suggest that the binding specificity in the C-terminal hinge region provides a great potential value for the development of new type-C inhibitors with highly selective and anti-resistant properties.

## Materials and Methods

### Preparations of kinase structures and screening libraries

A total of 51 protein kinase structures were selected from the Protein Data Bank (PDB) for site-moiety map constructions and virtual screening, and these include 45 tyrosine kinases and 6 kinases across the other families selected for the analyses (Table S1 in the supporting materials). These kinase structures were aligned to the EGFR structure (PDB code 3GT8) using a structural alignment tool^44^, and the alignments were then manually refined according to several conserved motifs, such as the DFG motif. These binding sites were defined by the residues situated at most 10 Å from the bound ligand ANP, an ATP analog, in the EGFR structure. The kinase-inhibitor (e.g., erlotinib and sorafenib) complex structures selected to compare with the type-C inhibitors were also aligned to the EGFR structure using the same procedure.

The kinase inhibitors used for constructing the site-moiety maps were collected from the BindingDB database^20^. First, 36,323 compounds that inhibit at least one human protein kinase with an IC_50_ or *K*_d_ value of less than 10 μM were selected. These compounds were then clustered through hierarchical clustering based on their topological features generated by the atom pair approach^45^. We finally yielded 4,680 inhibitors by selecting representative compounds from each cluster. Furthermore, we collected 118,759 natural compounds from the following seven vendors based on the ZINC compound database^24^: AnalytiCon Discovery NP, IBScreen NP, Indofine Natural Products, Molecular Diversity Preservation International, Princeton NP, Selleck BioChemicals NP, and Specs Natural Products. These natural compounds were screened for the identification of selective and broad-spectrum inhibitors.

### Constructions of specific and core site-moiety maps

The selected 4,680 kinase inhibitors and the 118,759 natural compounds were docked into the binding sites of the protein kinases using an in-house docking tool, GEMDOCK^21^. The docked poses of the top-ranked 2,000 kinase inhibitors were used to construct the site-moiety maps. For each kinase, we first generated interaction profiles to represent the interactions (i.e., electrostatic, hydrogen-bonding, or van der Waals interaction types) between the inhibitors and the kinase residues using the piecewise linear potential scoring function of GEMDOCK^21^. Each profile was represented by a binary matrix with size *P* × *C*, where *P* and *C* are the inhibitor number and the residue number of the kinase, respectively. A cell of this binary matrix was set to 1 if an inhibitor interacted with the residue; otherwise the cell was set to 0.

From the generated interaction profiles, the interacting residues were grouped into anchors based on the presence of consensus interactions (as derived by Z scores) and their binding pockets. For each interaction profile, the Z score (*Z*_*i*_) of residue *i* was calculated by 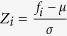, where *f*_*i*_ is the interaction frequency between the inhibitors and residue *i,* and *μ* and *σ* are the mean and standard deviation of an interaction frequency obtained from 1,000 randomly shuffled interaction profiles, respectively. The threshold for determining consensus interactions was set to 1.645, which corresponds to a 95% confidence level, a common threshold in statistics. The moiety preferences of each anchor were subsequently analyzed based on moieties defined by checkmol (http://merian.pch.univie.ac.at/~nhaider/cheminf/cmmm). Finally, the site-moiety maps of the protein kinases were constructed, and each anchor consists of a binding pocket with conserved interacting residues, moiety preferences, and interaction types.

Based on the three-dimensional anchor positions, we aligned these 51 site-moiety maps to infer the specific and core site-moiety maps. In these superimposed site-moiety maps, the anchors were considered the conserved alignment if the distance of their anchor centers was less than 2 Å^16^,^17^. These anchors were then divided into two specific and five core anchors (Fig. S1 in the supporting materials), which comprise the specific and core site-moiety maps, respectively. Based on the specific anchors, the selected kinases were divided into three groups: (1) Group 1 possessing the CHG and CH anchors, (2) Group 2 possessing only the CH anchor, and (3) Group 3 possessing only five core anchors. We then selected 9 representative kinases from each group (Group 1: EGFR, SYK, and IGF1R; Group 2: INSR, CSNK1G1, and ABL1; and Group 3: RET, STK24, and CDK5) for virtual screening to identify potential inhibitors. For each group, the 118,759 natural compounds were ranked by their total site-moiety map score. Finally, we selected compounds for the kinase profiling based on the total site-moiety map score, drug-like properties, availabilities, and domain knowledge for the kinase profiling.

### Kinase profiling

The natural compounds were purchased from Sigma-Aldrich. The natural compound profiling against the 64 protein kinases was conducted using the KinaseProfiler service offered by Millipore. The compounds were tested at a single concentration of 10 μM. The assay protocols used for the kinase profiling are available at http://www.eurofins.com/media/9724077/kinaseprofiler_assay_protocol_guide_eurofins_v64.pdf.

### Enzyme-based assay for wild-type and drug-resistant EGFR

To measure the activity of the compounds in inhibiting EGFR kinase activity, the expressions of wild-type (WT) and double-mutation (DM) EGFR kinases were determined as described previously^46^,^47^. Briefly, the expression constructs GST-EGFR-KDWT containing the kinase domain of human EGFR (amino acids 696–1,022) and GST-EGFR-KD L858R/T790M containing the kinase domain of EGFR with the T790M and L858R mutations were expressed in Sf9 insect cells. The expression constructs were co-transfected with linear BacPAK8 viral DNA into Sf9 insect cells. The culture medium from baculovirus-infected insect cell cultures was harvested and collected as virus stocks for the production of an EGFR-related kinase following standard protocols. Recombinant baculoviruses, namely Bac-EGFR^KD-WT^, Bac-EGFR^KD-T790M^, Bac-EGFR^KD-L858R^, and Bac-EGFR^KD-T790M/L858R^ were generated to express wild-type and the T790M, L858R and T790M/L858R mutants of the EGFR kinase domain. GST-fusion proteins were then expressed in Sf9 insect cells and purified according to the manufacturer’s instructions.

Kinase assays were performed in a final volume of 50 μL with the following components: 100 to 300 ng of EGFR in 50 mM HEPES, pH 7.4, 10 mM MgCl2, 10 mM NaCl, 1 mM DTT, 0.01% bovine serum albumin (BSA), 0.5 mM Na3VO4, and 1 μM ATP, 5 to 10 μM poly(Glu,Tyr) 4:1 and various concentrations of RA and RA analogs. The reactions were then conducted for 2 h at 30 °C. After the incubation, 50 μL of the Kinase-Glo Plus reagent (Promega) was added to the reactions, and the mixture was then incubated for 20 min at 25 °C. The luminescence was measured with a Wallac Vector 1420 multilabel counter (PerkinElmer, Wallac, Turku, Finland).

### Synthesis of rosmarinic acid derivatives

The rosmarinic acid derivatives were synthesized using the procedures described in the supporting materials (Figs. S7, S8, S9, and S10 in the supporting materials).

### Invasion assay

The invasion assay was conducted using the cell-based assay service offered by Reaction Biology Corporation. In brief, MDA-MB-231 breast cancer cells cultured for 1 day were serum-starved overnight. The cells were then collected and resuspended (2 × 10^5^ cells) in 500 μL of serum-free DMEM medium. Culture inserts (Corning BioCoat Matrigel invasion chambers) were placed in the wells of a Corning BioCoat Matrigel Invasion plate, and 750 μL of DMEM with 20% serum was added to the lower compartment of each well. Cell suspensions were added to each culture insert. The cells were incubated for 22 h. The excess cells were then removed with a cotton swab from the top of the chamber, and the invaded cells were fixed in methanol for 10 min and stained with toluidine blue for 5 min. The invaded cells were then photographed under a microscope at a magnification of approximately 4X and the cell density was counted using the Image Parsing software. Finally, the percentage of invaded cells was calculated by subtracting the cell number of the control group to measure the invasiveness of the cancer cells.

### Statistical analysis

Results of the invasion assay are expressed as the mean ± standard error of the mean. Statistical analysis was performed by a one-way ANOVA with Tukey’s multiple comparison test. A *p* value of 0.05 or less was considered statistically significant.

## Additional Information

**How to cite this article**: Hsu, K.-C. *et al.* Anchor-based classification and type-C inhibitors for tyrosine kinases. *Sci. Rep.* **5**, 10938; doi: 10.1038/srep10938 (2015).

## Supplementary Material

Supplementary Information
