# Supplementary material for: Anchor-based classification and type-C inhibitors for tyrosine kinases
Source: Sci Rep. 2015 Jun 16;5:10938. doi: 10.1038/srep10938 (PMC4468516; doi:10.1038/srep10938)
Supplement: Supplementary Information [file srep10938-s1.pdf]

Anchor-based classification and type-C inhibitors for tyrosine kinases

Kai-Cheng Hsu[1], Tzu-Ying Sung[1], Chih-Ta Lin[1], Yi-Yuan Chiu[1], John T.-A. Hsu[2, 3], Hui-Chen Hung[3], Chung-Ming Sun[4], Indrajeet Barve[4], Wen-Liang Chen[2], Wen-Chien Huang [5], Chin-Ting Huang[3], Chun-Hwa Chen[3], and Jinn-Moon Yang[1,2*]

[1]Institute of Bioinformatics and Systems Biology, National Chiao Tung University, Hsinchu, Taiwan

[2]Department of Biological Science and Technology, National Chiao Tung University, Hsinchu, Taiwan

[3]Institute of Biotechnology and Pharmaceutical Research, National Health Research Institutes, Miaoli, Taiwan

[4]Department of Applied Chemistry, National Chiao Tung University, Hsinchu, Taiwan

[5]Department of Thoracic Surgery, Mackay Memorial Hospital, Taipei City, Taiwan

*Corresponding authors

Core anchors of protein kinases in ATP-binding site

The five core anchors, which represent the common characteristics of the protein kinases, are named the pyrimidine ring of adenine (PA), the imidazole of adenine (IA), ribose (RB), α-phosphate (AP), and β-phosphate (BP) anchors, according to their locations (Fig. S2A in the supporting materials). We used the residue numbering of EGFR as the reference for describing the anchors. The PA anchor consists of the residues L718, V726, Q791, M793, and L844 and has two preferred interaction types: hydrogen-bonding and van der Waals interactions. This anchor strongly preferentially interacts with ring moieties containing polar atoms, such as heterocyclic groups, phenols, and adenine. Such polar rings can be sandwiched by the long side chains of the three hydrophobic residues L718, V726, and L844, whereupon the polar atoms of the rings yield hydrogen bonds with the other two residues, Q791 and M793, on the hinge. For example, the adenine of ANP (ATP analog) located in the PA anchor forms van der Waals interactions with the residues L718, V726, and L844 and forms hydrogen bonds with the residues Q791 and M793 (Fig. S2A in the supporting materials). The IA anchor is close to the PA anchor and consists of the interacting residues V726, T790, L844, and T854. These two anchors have similar moiety preferences (Fig. S2B in the supporting materials). Many known kinase inhibitors and drugs (e.g., gefitinib and erlotinib) are designed with polar ring moieties to mimic the adenine of ATP in these two anchors.

The RB anchor, which is situated in the ribose-binding region (Fig. S2A in the supporting materials), consists of two interacting residues, G719 and V726. The most abundant interactions are van der Waals interactions formed by ring moieties or bulky groups, such as aromatic rings, heterocyclic moieties, and alkenes. The co-crystal structure of the EGFR

complexed with ANP indicates that the interacting residues of the anchor produce van der Waals interactions with the ribose of ATP. Two remaining anchors, namely AP and BP, are located adjacent to the α-phosphate and β-phosphate moieties of ATP, respectively (Fig. S2A in the supporting materials). The AP anchor consists of conserved interacting residues V726, K745, and D855, and the BP anchor consists of interacting residues K745, D837, N842, and D855 (Fig. S2A in the supporting materials). The residues K745 and N842 generate hydrogen bonds with the phosphate moieties of ATP and play an important role in the catalytic process. The former is the catalytic residue for phosphate transfer [1], whereas the latter is one of the critical residues for ATP binding [2]. Mutations at the 745 position result in the loss of kinase activity [3]. The AB and BP anchors consist of residues with polar and long side chains and prefer moieties that simultaneously yield hydrogen bonds and van der Waals interactions, such as sulfuric acid and phosphoric acid derivatives (Fig. S2B in the supporting materials). These five core anchors are conserved in protein kinases because they play important roles in the ATP binding process. Additionally, the compounds that only match the core anchors are often broad-spectrum inhibitors that affect most protein kinases. These results show that our site-moiety maps can identify the conserved interacting residues and the preferred moiety to reflect the ATP binding mechanisms and protein kinase functions.

Groups of anchor-based classification of tyrosine kinases

Several kinases are classified as Group 2 instead of Group 1, even though the residues of their CHG anchor are similar or conserved. This is because the Group 2 residue located at the 718 position on the G-loop shifts toward the C-terminal hinge region, which eliminates the CHG anchor pocket. For example, EGFR and INSR are classified into Groups 1 and 2, respectively, even though these two kinases belong to the same family and share a sequence identity of 37% (Fig. S1 in the supporting materials). We further compared the structures of EGFR and INSR (Fig. S4A in the supporting materials). Although most of the anchor residues are conserved between EGFR and INSR in the CHG anchor (L718, M793, and G796 in EGFR; L1002, M1079, and G1082 in INSR, respectively), the spatial positions of residues L718 and L1002 are different (Fig. S4A in the supporting materials). The distance between residue 718 on the G-loop and the C-terminal hinge is shorter in INSR than in EGFR, which results in INSR being unable to form a binding pocket for accommodating compound moieties.

There are two reasons for the elimination of the CH anchor in Group 3. First, some kinases (e.g., RET and EPHA7) have short side-chain residues (i.e., A/G/S/T) at positions 797 and 800 (Figs. 2C and S3C in the supporting materials); hence, these residues are unable to stabilize van der Waals interactions with compounds. Second, the side chains of the other kinases (e.g., CDK5 and STK24) at these two positions are long and pointed toward the pocket center (Fig. 2C), eliminating this pocket. For example, residues Q85, D86, and K89 of CDK5-CMGC (corresponding to G796, C797, and D800 in EGFR-TK, respectively) have longer side-chains than EGFR-TK (Fig. S4B in the supporting materials). Of these residues, Q85 and K89 are pointed toward the pocket center, and occupy the binding pocket of the anchor. Another example is residue D109 in STK24-STE, which corresponds to residue D800 in EGFR-TK, similarly to the way in which residue K89 functions in the CDK5-CMGC kinase. Its side chain is oriented toward the pocket center, which eliminates the anchor (Fig. S4B in the supporting materials). These observations suggest that the side-chain orientation of the residue at the position 800 plays a key role in forming the CH anchor.

These two specific anchors in the C-terminal hinge region can be used to design type-C inhibitors for the kinases in Groups 1 and 2. The anchor residues of the CHG anchor often form van der Waals interactions with aromatic rings, heterocyclic moieties, phenols, alkenes, and enamines (Figs. S2C and S2D in the supporting materials). For example, the heterocyclic moieties can fit the CHG pocket well with an average energy of -20.3 kcal/mol statistically derived from more than 100 compounds. The major interacting moieties of the CH anchor are aromatic rings, heterocyclic moieties, enamines, and amides, which often form van der Waals or hydrogen-bonding interactions (Fig. 2B). These moieties can form stable interactions with the anchor residues and can be used for guiding lead optimization.

Binding mode of quercetin

Quercetin showed the broadest protein kinase inhibitory activity in the kinase profiling assay because it matches three core anchors (PA, IA, and RB) without any specific anchors (Fig. 3G). The predicted quercetin-EGFR complex indicates that the dihydroxychromone group of quercetin occupies the location occupied by the adenine of ATP. The two hydroxyl moieties of quercetin form three hydrogen bonds with the main chains of the anchor residues T790, Q791, and M793. The planar ring of dihydroxychromone makes van der Waals contacts with the hydrophobic residues L718, V726, and L844 in the PA and IA anchors. The

pyrocatechol moiety of quercetin is located in the ribose-binding region and forms van der Waals interactions with residues G719 and V726 of the RB anchor in a similar way to the ribose of ATP. Because quercetin only matches the core anchors at the ATP binding site conserved in all protein kinases, it is often able to bind most kinases without selection, such as INSR and RET (Figs S5E and S5F in the supporting materials).

Experimental procedure for synthesizing rosmarinic acid derivatives

RA-D1

Rosmarinic acid (0.05 g, 0.13 mmol) was dissolved in DMF (20 mL). EDCI (0.028 g, 0.18 mmol) and HOBT (0.02 g, 0.15 mmol) were added at 0°C, and the reaction mixture was stirred for 10 min. To the above reaction mixture, 2-methoxy-ethyl-amine (0.011 g, 0.15 mmol) was added, and the reaction mixture was stirred at room temperature for 16 h. After completion of the reaction, the reaction mixture was diluted with water (20 mL) and extracted with ethyl acetate (3 x 25 mL). The combined organic layers were washed with brine (30 mL), dried over $MgSO_4$ and concentrated *in vacuo*. The crude product was dissolved in dichloromethane (15 mL) and re-precipitated through the dropwise addition of hexane (15 mL). The precipitated solid product was filtered and washed with hexane (30 mL) to afford RA-D1 (0.05 g, 86%). ^{1}H NMR (400 MHz, acetone) δ 7.54 (d, *J* = 15.9 Hz, 1H), 7.21 (s, 1H), 7.15 (d, *J* = 2.1 Hz, 1H), 7.04 (dd, *J* = 8.2, 2.0 Hz, 1H), 6.87 (d, *J* = 8.2 Hz, 1H), 6.77 (d, *J* = 2.0 Hz, 1H), 6.72 (d, *J* = 8.0 Hz, 1H), 6.59 (dd, *J* = 8.0, 2.0 Hz, 1H), 6.29 (d, *J* = 15.9 Hz, 1H), 5.28 (dd, *J* = 7.4, 4.7 Hz, 1H), 3.39 – 3.27 (m, 4H), 3.24 (s, 3H), 3.06 (dd, *J* = 14.1, 4.7 Hz, 1H), 2.98 (dd, *J* = 14.1, 7.4 Hz, 1H); ^{13}C NMR (101 MHz, acetone) δ 169.14, 165.54, 148.03, 145.57, 145.41, 144.69, 143.77, 128.26, 126.60, 121.79, 120.91, 116.54, 115.47, 114.92, 114.35, 114.18, 74.32, 70.71, 57.70, 38.51, 37.20; MS (ESI+) *m/z*: 440.2 $(M+Na)^+$.

RA-D2

Rosmarinic acid (0.05 g, 0.13 mmol) was dissolved in DMF (20 mL). EDCI (0.028 g, 0.18 mmol) and HOBT (0.02 g, 0.15 mmol) were added at 0°C, and the reaction mixture was stirred for 10 min. Isopropyl amine (0.009 g, 0.15 mmol) was added. The reaction mixture was stirred at room temperature until the starting materials were consumed (16 h). The reaction mixture was then diluted with water (20 mL) and extracted with ethyl acetate (3 x 25 mL). The combined organic layers were washed with brine (30 mL), dried over $MgSO_4$ and

concentrated *in vacuo*. The crude product was dissolved in dichloromethane (15 mL) and re-precipitated through the dropwise addition of hexane (15 mL). The precipitated solid product was filtered and washed with hexane (30 mL) to afford RA-D2 (0.051 g, 92 %). ^{1}H NMR (300 MHz, acetone) δ 8.12 (s, 2H), 7.54 (d, *J* = 15.9 Hz, 1H), 7.16 (s, 2H), 7.04 (d, *J* = 8.1 Hz, 1H), 6.94 – 6.83 (m, 1H), 6.78 (s, 1H), 6.73 (d, *J* = 7.9 Hz, 2 H), 6.60 (d, *J* = 8.0 Hz, 1H), 6.29 (d, *J* = 15.9 Hz, 1H), 5.22 (t, *J* = 6.0 Hz, 1H), 4.13 – 3.90 (m, 1H), 3.20 – 2.87 (m, 3H), 1.07 (d, *J* = 6.3 Hz, 6 H); ^{13}C NMR (101 MHz, acetone) δ 168.78, 165.79, 148.16, 145.68, 145.49, 144.78, 143.84, 128.08, 126.51, 121.83, 120.91, 116.63, 115.50, 114.97, 114.36, 114.09, 74.46, 41.00, 37.28, 21.67, 21.63.; MS (ESI+) *m/z*: 402.3 $(M+H)^+$.

RA-D3

Rosmarinic acid (0.05 g, 0.13 mmol) was dissolved in DMF (20 mL) and EDCI (0.028 g, 0.18 mmol), HOBT (0.02 g, 0.15 mmol) were added at 0°C. After 10 min of stirring at 0°C, cyclopentyl amine (0.012 g, 0.15 mmol) was added, and the mixture was stirred at room temperature until the disappearance of the starting materials (16 h). The reaction mixture was then diluted with water (20 mL) and extracted with ethyl acetate (3 x 25 mL). The combined organic layers were washed with brine (30 mL), dried over $MgSO_4$ and concentrated *in vacuo*. The crude product was dissolved in dichloromethane (15 mL) and re-precipitated through the dropwise addition of hexane (15 mL). The precipitated solid product was filtered and washed with hexane (30 mL) to afford RA-D3 (0.052 g, 89%). ^{1}H NMR (400 MHz, acetone) δ 7.53 (d, *J* = 16.0 Hz, 1H), 7.15 (s, 1H), 7.08 – 6.99 (m, 2H), 6.87 (d, *J* = 8.2 Hz, 1H), 6.76 (s, 1H), 6.72 (d, *J* = 8.0 Hz, 1H), 6.58 (d, *J* = 8.0 Hz, 1H), 6.28 (d, *J* = 15.9 Hz, 1H), 5.20 (t, *J* = 6.1 Hz, 1H), 4.19 – 4.08 (m, 1H), 3.03-2.96 (m, 2 H), 1.91 – 1.76 (m, 2H), 1.67 – 1.43 (m, 4H), 1.42 – 1.34 (m, 2H); ^{13}C NMR (101 MHz, acetone) δ 168.62, 165.68, 148.02, 145.46, 145.42, 144.72, 143.75, 128.26, 126.60, 121.77, 120.88, 116.57, 115.47, 114.91, 114.36, 114.27, 74.47, 50.71, 50.59, 37.25, 32.24, 32.19, 23.46; MS (ESI+) *m/z*: 428.2 $(M+H)^+$.

RA-D4

Caffeic acid (0.05 g, 0.27 mmol) was dissolved in DMF (20 mL). EDCI (0.028 g, 0.18 mmol) and HOBT (0.02 g, 0.15 mmol) were added at 0°C, and

the mixture was stirred for 10 min. Dopamine hydrochloride (0.057 g, 0.30 mmol) was added to the reaction mixture. The reaction mixture was stirred at room temperature for 16 h. After the reaction was completed, the resulting mixture was diluted with water (20 mL) and extracted with ethyl acetate (3 x 25 mL). The combined organic layers were washed with brine (30 mL), dried over $MgSO_4$ and concentrated *in vacuo*. The crude product was dissolved in dichloromethane (15 mL) and re-precipitated through the dropwise addition of hexane (15 mL). The precipitated solid product was filtered and washed with hexane (30 mL) to afford RA-D4 (0.078 g, 89%).^{1}H NMR (300 MHz, acetone) δ 7.41 (d, *J* = 15.7 Hz, 1H), 7.21 (s, 1H), 7.07 (s, 1H), 6.94 (d, *J* = 8.2 Hz, 1H), 6.84 (d, *J* = 8.1 Hz, 1H), 6.74 (d, *J* = 7.4 Hz, 2H), 6.57 (d, *J* = 7.8 Hz, 1H), 6.44 (d, *J* = 15.6 Hz, 1H), 3.53 – 3.42 (m, 2H), 2.69 (t, *J* = 7.2 Hz, 2H); ^{13}C NMR (101 MHz, CD_3OD) δ 167.84, 140.71, 134.63, 130.68, 120.65, 119.60, 115.44, 115.01, 114.96, 113.45, 41.11, 34.63; MS (ESI+) *m/z*: 316.2 $(M+H)^+$.

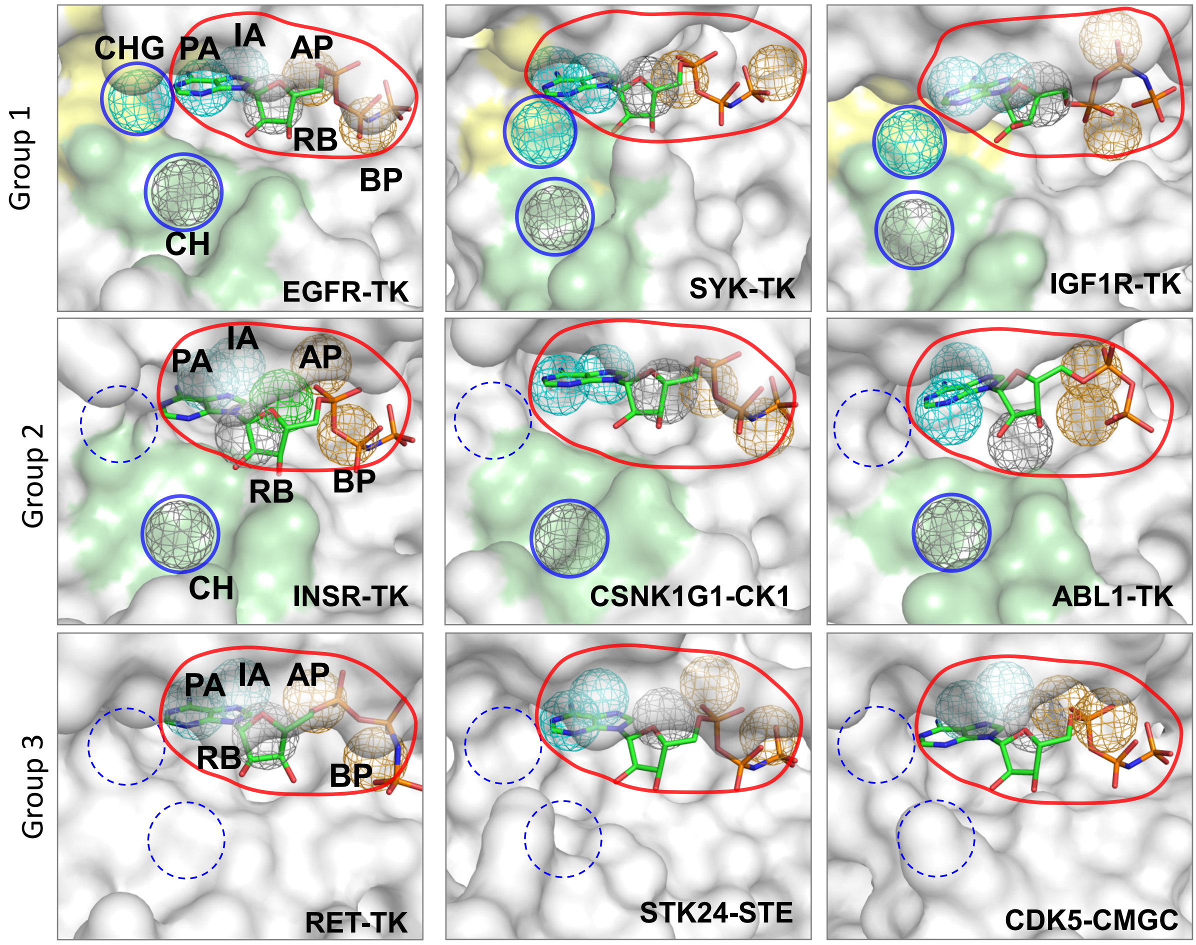

Figure S1. Site-moiety maps of three anchor-based groups. The anchors of the site-moiety maps of EGFR-TK (PDB 3GT8), SYK-TK (PDB 3EMG), IGF1R-TK (PDB 1JQH), INSR-TK (PDB 1IR3), CSNK1G1-CK1 (PDB 2CMW), ABL1-TK (PDB 2G2I), RET-TK (PDB 2VIT), STK24-STE (PDB 3A7J), and CDK5-CMGC (PDB 1UNL) are represented by mesh spheres. The bound ligand of EGFR (ANP) is shown as a reference. The tyrosine kinases have two specific anchors (blue circle) and five core anchors (red circle). Group 1 kinases have two specific anchors; Group 2 kinases have only the CH anchor; and Group 3 kinases lack both specific anchors.

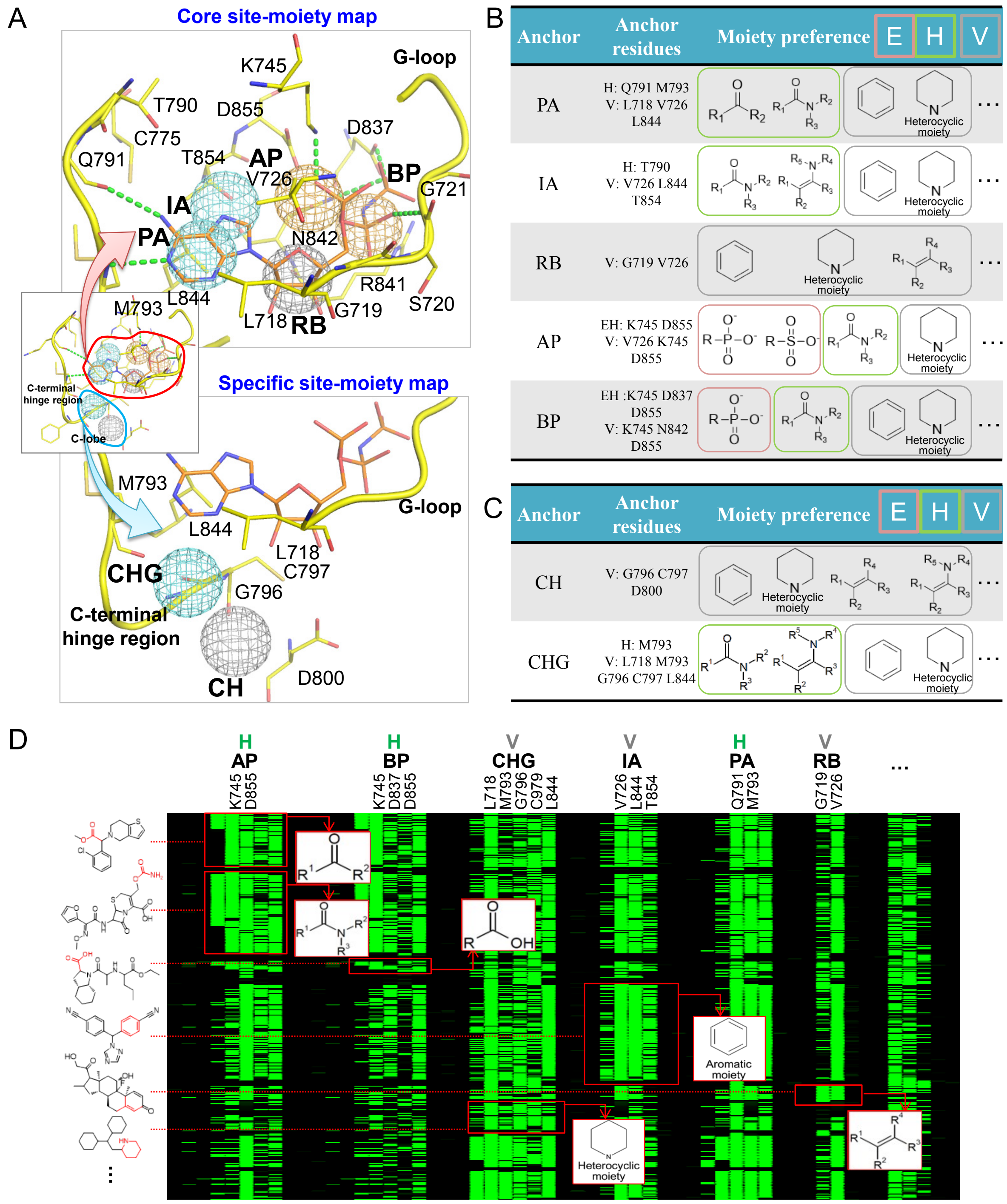

Figure S2. Specific and core site-moiety maps of tyrosine kinases. (A) Specific and conserved anchors (mesh spheres) located in the C-terminal hinge region and the ATP-binding site, respectively. ANP (ATP analog), a ligand of EGFR, is shown as a reference (PDB code 3GT8). Hydrogen bonds between the anchor residues and ANP are represented by green dashed lines. Anchor residues, moiety preferences, and interaction types of the (B) core and (C) specific anchors. The interaction types include electrostatic (E), hydrogen bonding (H), and van der Waals (V) interactions. Anchors with three (E, H, and V), two (H and V) and one (V) interaction type are colored cyan, orange, and grey, respectively. (D) Compound-residue interaction profile. An entry is colored green if the compound interacts with the residue; otherwise, the entry is colored black. The interaction profile is used to generate anchors, including conserved interacting residues, moiety preferences, and interaction types.

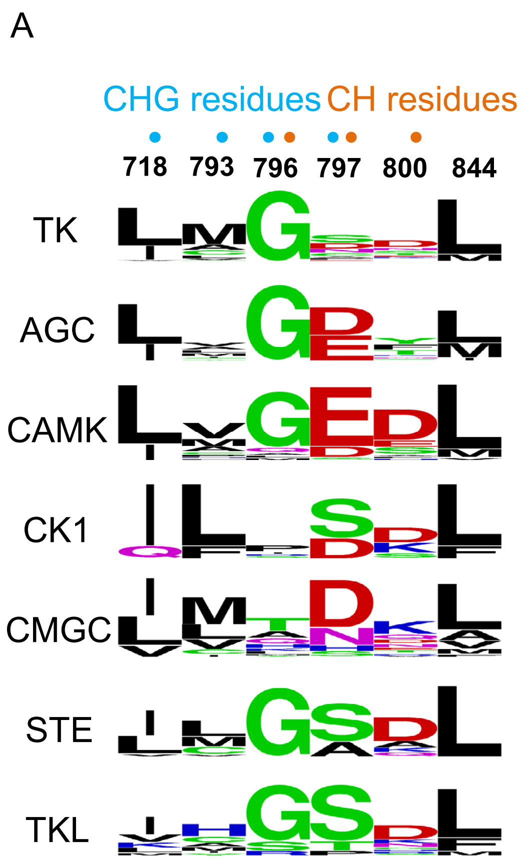

B

Group (anchor)	Family	Kinase	CHG	CH	718	792	793	796	797	800	844
1	TK	EGFR			L	L	M	G	C	D	L
1	TK	ERBB4			L	L	M	G	C	E	L
1	TK	ERBB2			L	L	M	G	C	D	L
1	TK	TNK2			L	L	A	G	S	D	L
1	TK	SYK			L	M	A	G	P	K	L
1	TK	ZAP70			L	M	A	G	P	K	L
1	TK	IGF1R			L	L	M	G	D	S	M
1	TK	ALK			L	L	M	G	D	S	L
2	TK	INSR			L	L	M	G	D	S	M
2	TK	PTK2			I	L	C	G	E	S	L
2	TK	FES			I	L	V	G	D	T	L
2	TK	KIT			L	Y	C	G	D	N	L
2	TK	FLT3			L	Y	C	G	D	N	L
2	TK	CSF1R			L	Y	C	G	D	N	L
2	TK	MERTK			L	F	M	G	D	T	M
2	TK	MET			I	Y	M	G	D	N	M
2	TK	MST1R			I	Y	M	G	D	Q	M
2	TK	HCK			L	F	M	G	S	D	L
2	TK	CSK			I	Y	M	G	S	D	L
2	TK	LCK			L	Y	M	G	S	D	L
2	TK	JAK2			L	Y	L	G	S	D	L
2	TK	FYN			L	Y	M	G	S	D	L
2	TK	ABL1			L	F	M	G	N	D	L
2	TK	SRC			L	Y	M	G	S	D	L
2	TK	JAK1			L	F	L	G	S	E	L
2	TK	ABL2			L	Y	M	G	N	D	L
2	TK	ERBB3			L	Y	L	G	S	D	L
2	TK	TEK			I	Y	A	G	N	D	L
2	TK	FGFR2			L	Y	A	G	N	E	L
2	TK	FGFR1			L	Y	A	G	N	E	L
2	TK	LYN			L	Y	M	G	S	D	L
2	TK	JAK3			L	Y	L	G	C	D	L
2	TK	ITK			I	F	M	G	C	D	L
2	TK	BTK			L	Y	M	G	C	N	L
2	TK	KDR			L	F	C	G	N	T	L
2	TK	FLT1			L	Y	C	G	N	N	L
2	TK	TYK2			L	Y	V	G	P	V	L
2	TK	EPHA2			I	Y	M	G	A	K	L
2	TK	PTK2B			L	L	Y	G	E	H	L
2	CK1	CSNK1G1			I	L	L	P	S	D	L
3	TK	RET			L	Y	A	G	S	G	L
3	TK	EPHA3			V	Y	M	G	S	S	L
3	TK	EPHA5			I	Y	M	G	S	T	L
3	TK	EPHA8			I	Y	M	G	S	T	L
3	TK	EPHB4			I	F	M	G	A	S	L
3	TK	EPHA7			I	F	M	G	A	A	L
3	TKL	LIMK1			L	Y	I	G	T	G	L
3	STE	STK24			I	Y	L	G	S	D	L
3	AGC	RPS6KA1			L	F	L	G	D	T	L
3	CMGC	CDK5			I	F	C	Q	D	K	L
3	CAMK	PIM1			L	R	P	Q	D	D	L

Figure S3. Sequence analysis of specific anchors. (A) Anchor residue profiles of the seven kinase families using the residue numbering of EGFR as a reference. Residues at the 797 and 800 positions are more diverse in TK than the other kinase families, suggesting an opportunity to design TK-specific inhibitors. (B) Kinase-anchor profile and anchor residues of kinases. An anchor entry is colored green if the kinase has the anchor; otherwise, the entry is colored black. The anchor-based classification can distinguish specific characteristics from proteins even if they have similar sequences.

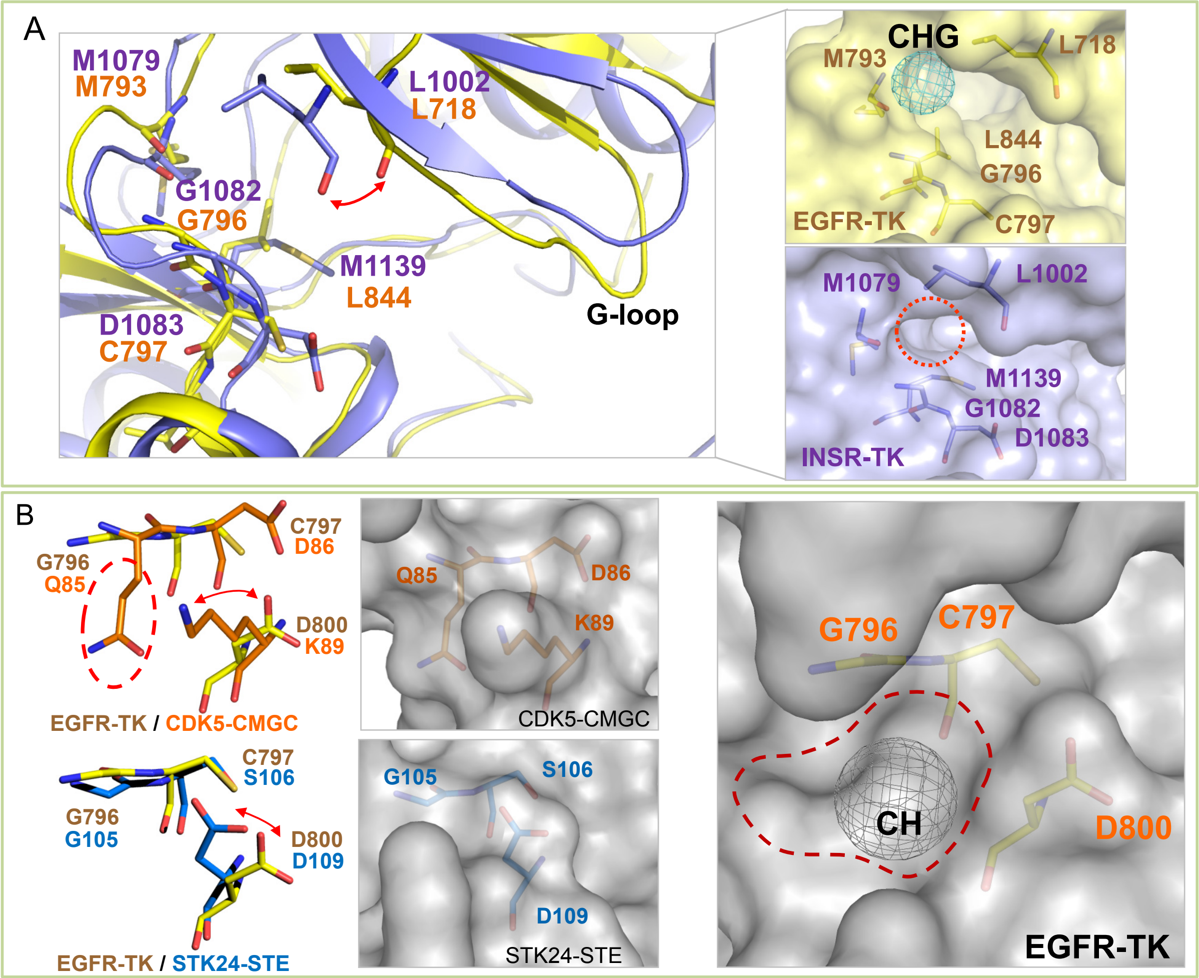

Figure S4. **Examples of structure comparisons between three group kinases.** (A) Comparison of the CHG anchor residues between the kinases EGFR (yellow, Group 1) and INSR (purple, Group 2). These two kinases are in the same tyrosine kinase family and share a sequence identity of 37%. In INSR, residue L1002 is much closer to the hinge residues (M1079 and G1082) than residue L718 of EGFR, which results in a relatively small binding pocket and elimination of the CHG anchor in INSR. (B) Comparison of the CH anchor residues between the kinases EGFR (yellow, Group 1), CDK5 (orange, Group 2), and STK24 (blue, Group 3). The comparison shows that the short side-chain residue at position 796 and the long side-chain residue at position 800 are essential for the formation of the binding pocket. In CDK5, residue Q85 with a long side chain causes the elimination of the CH anchor. In addition, the long side chain that is pointed toward the pocket center limits the formation of the binding pocket, as observed in STK24.

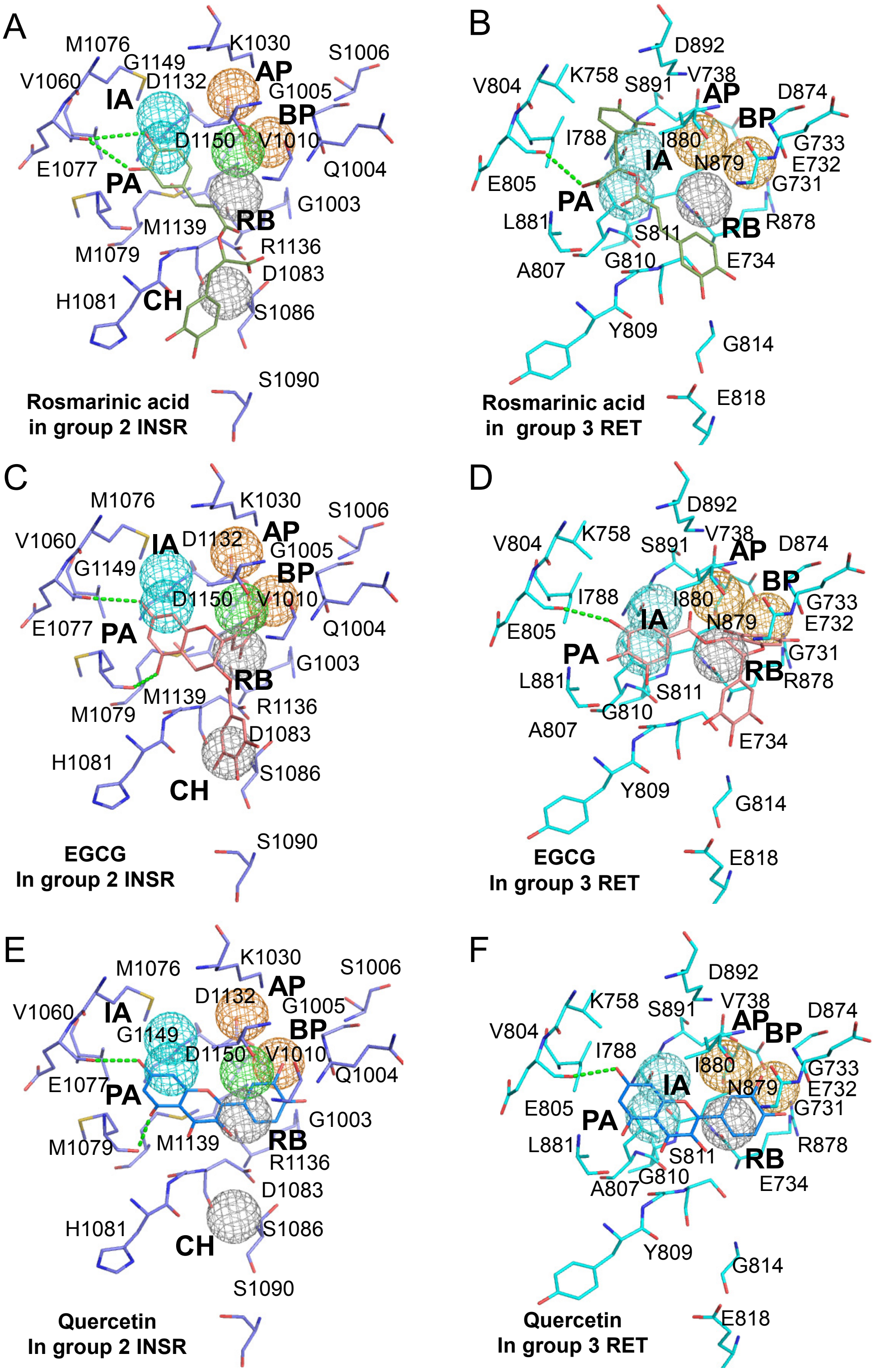

Figure S5. Docked poses of the compounds (A, B) rosmarinic acid, (C, D) EGCG, and (E, F) quercetin in the kinases INSR and RET. Rosmarinic acid is unable to simultaneously form interactions with both specific anchors of INSR and RET. EGCG matches the CH anchor of INSR but not of RET. Quercetin matches the core anchors of INSR (PDB code 1IR3) and RET (PDB code 2IVT). Hydrogen-bonding interactions are represented as green dashes.

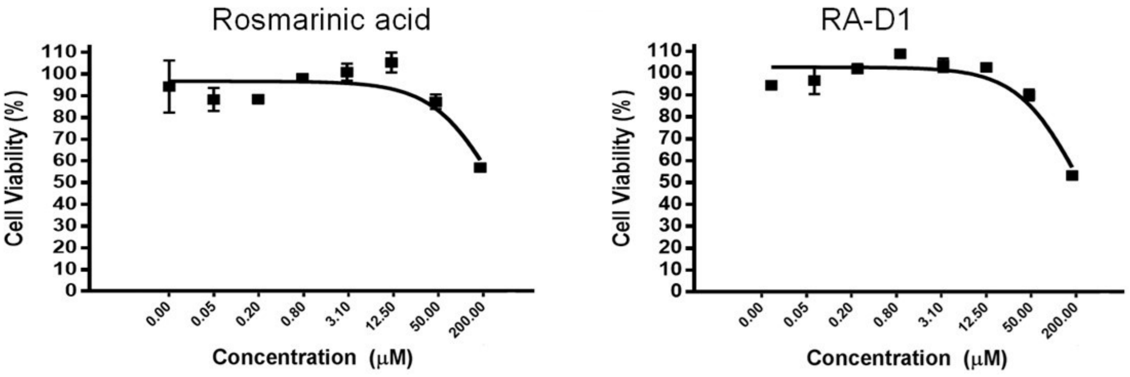

Figure S6. **Cell viability after treatment with various concentrations of type-C inhibitors.** The CellTiter-Glo Luminescent Cell Viability Assay was used to assess the cell viability for 22 h. The percentage of cell viability was calculated by subtracting the cell number of the control group.

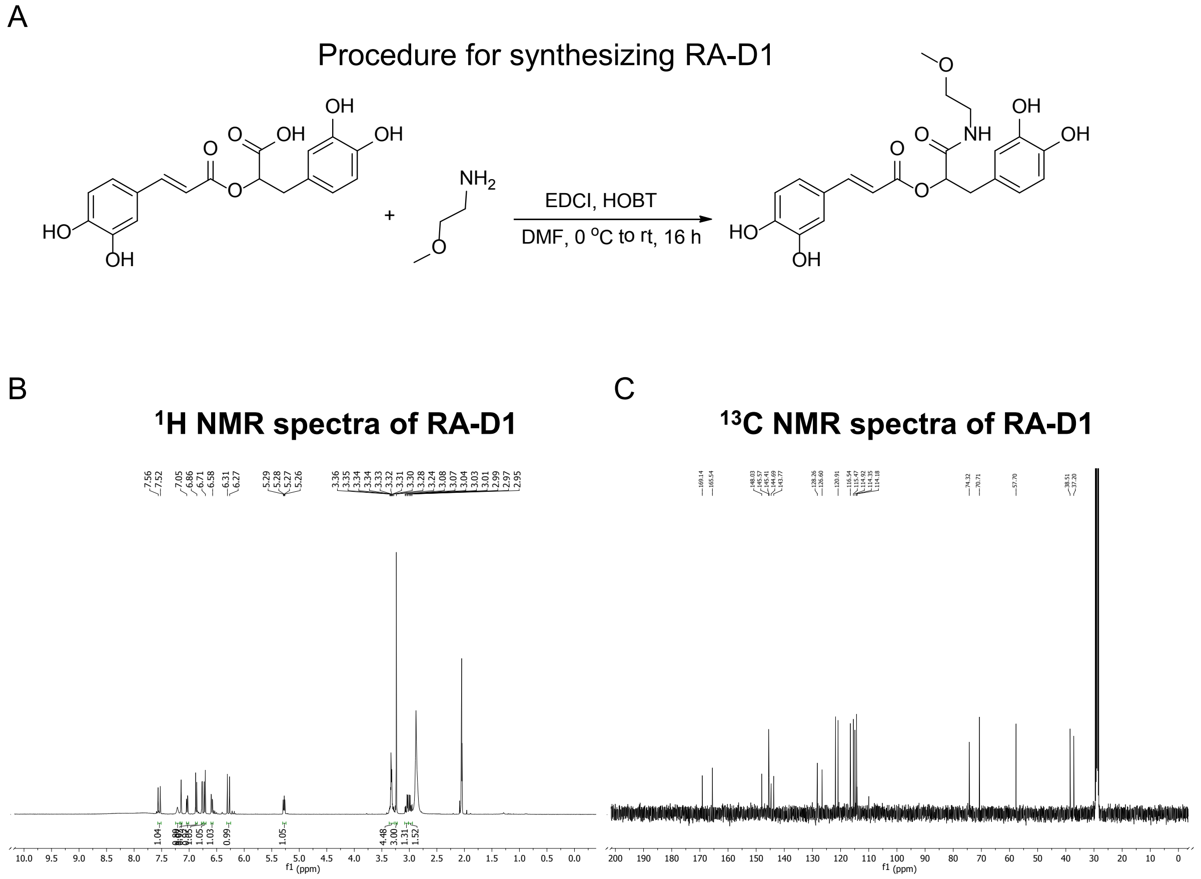

Figure S7. Synthesis of RA-D1. (A) Reaction scheme for synthesizing RA-D1. (B) ^{1}H NMR spectra of RA-D1. (C) ^{13}C NMR spectra of RA-D1.

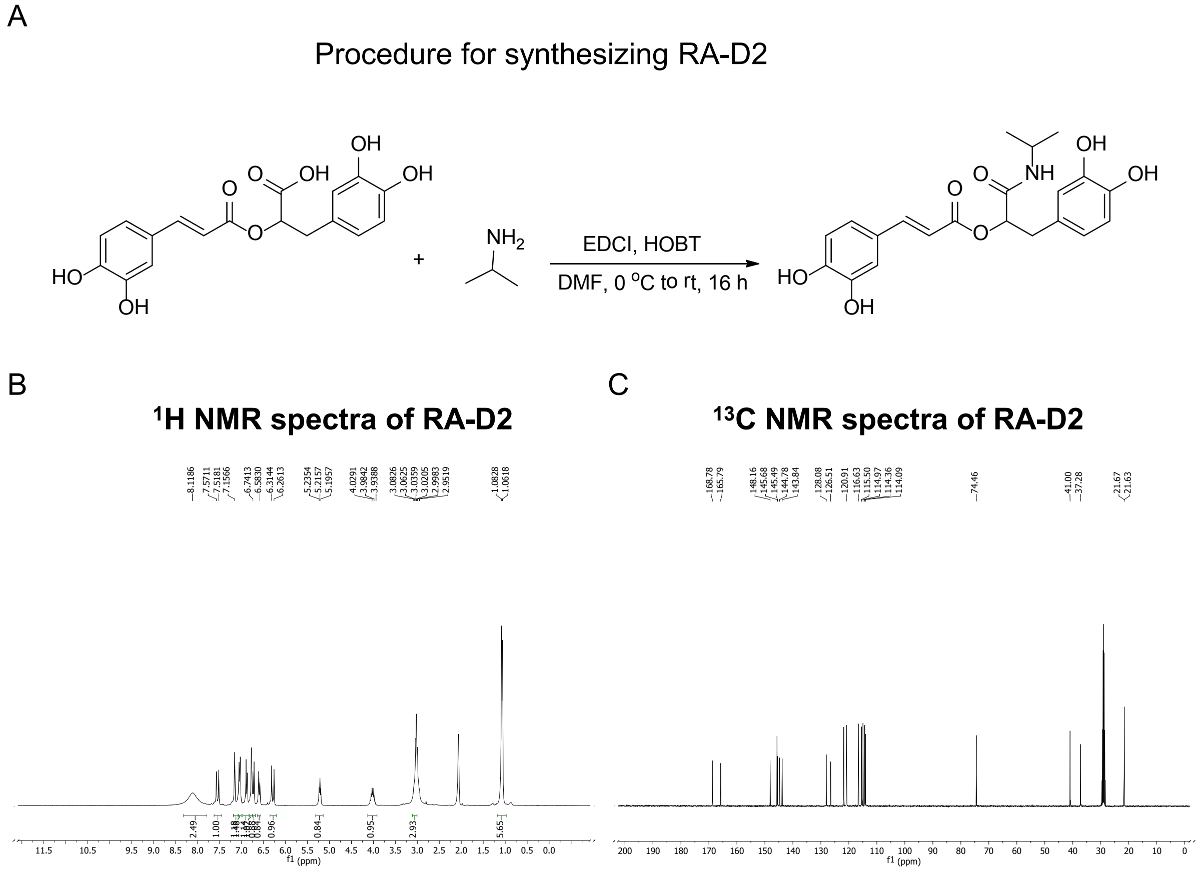

Figure S8. Synthesis of RA-D2. (A) Reaction scheme for synthesizing RA-D2. (B) ^{1}H NMR spectra of RA-D2. (C) ^{13}C NMR spectra of RA-D2.

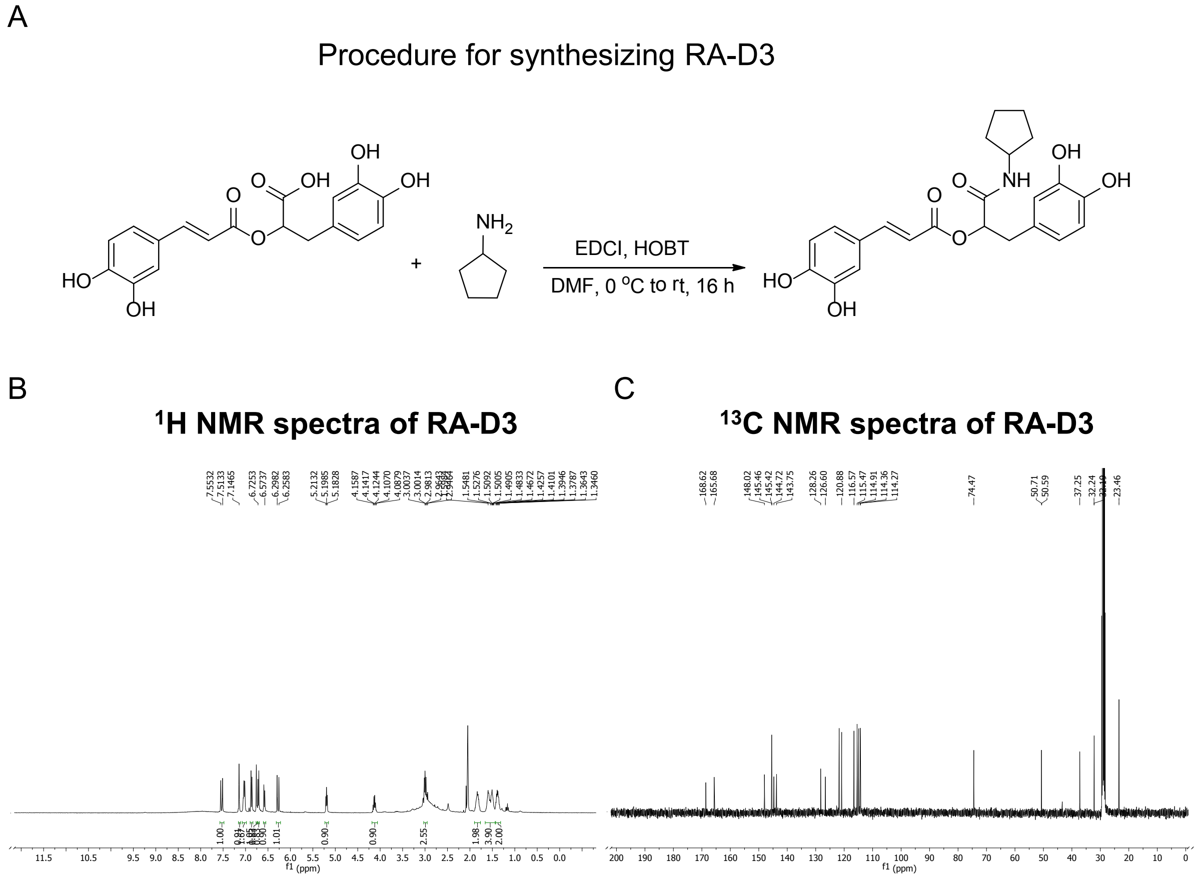

Figure S9. Synthesis of RA-D3. (A) Reaction scheme for synthesizing RA-D3. (B) ^{1}H NMR spectra of RA-D3. (C) ^{13}C NMR spectra of RA-D3.

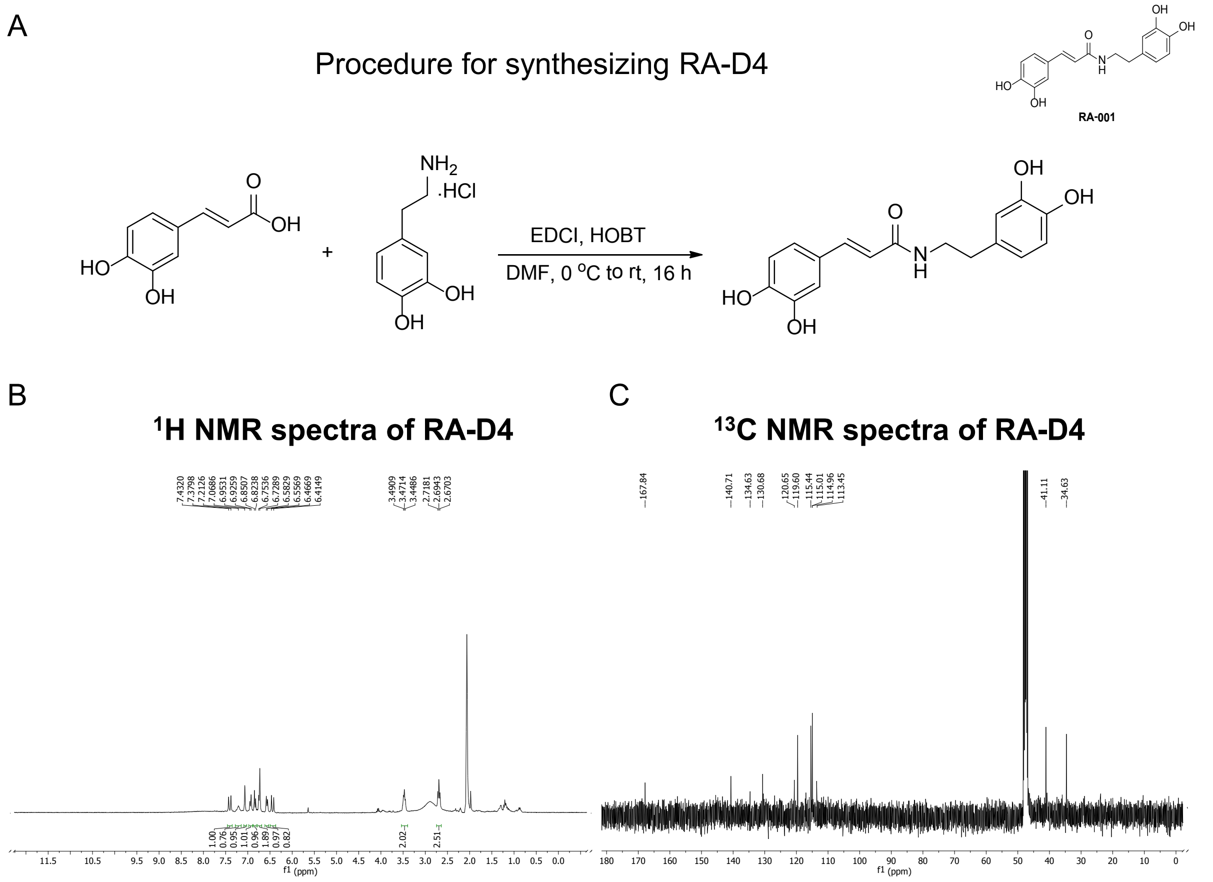

Figure S10. Synthesis of RA-D4. (A) Reaction scheme for synthesizing RA-D4. (B) ^{1}H NMR spectra of RA-D4. (C) ^{13}C NMR spectra of RA-D4.

Table S1. Selected protein kinases for analyses

Family	Kinase	PDB	Group	Family	Kinase	PDB	Group
TK	EGFR	3GT8	1	TK	ABL2	3KEX	2
TK	ERBB4	3BBT	1	TK	ERBB3	2OO8	2
TK	ERBB2	3PP0	1	TK	TEK	2PVF	2
TK	TNK2	1U54	1	TK	FGFR2	3RHX	2
TK	SYK	3EMG	1	TK	FGFR1	3A4O	2
TK	ZAP70	1U59	1	TK	LYN	1YVJ	2
TK	IGF1R	1JQH	1	TK	JAK3	1SM2	2
TK	ALK	3LCS	1	TK	ITK	3OCT	2
CK1	CSNK1G1	1IR3	2	TK	BTK	3BE2	2
TK	INSR	1MP8	2	TK	KDR	3HNG	2
TK	PTK2	3BKB	2	TK	FLT1	3LXP	2
TK	FES	1T45	2	TK	TYK2	1MQB	2
TK	KIT	1RJB	2	TK	EPHA2	3CC6	2
TK	FLT3	2I0Y	2	TK	PTK2B	2CMW	2
TK	CSF1R	2P0C	2	AGC	RPS6KA1	2IVT	3
TK	MERTK	3DKC	2	CAMK	PIM1	2QO7	3
TK	MET	3PLS	2	CMGC	CDK5	2R2P	3
TK	MST1R	1AD5	2	STE	STK24	3KUL	3
TK	HCK	1BYG	2	TK	RET	2VWU	3
TK	CSK	1QPC	2	TK	EPHA3	2REI	3
TK	LCK	2B7A	2	TK	EPHA5	3S95	3
TK	JAK2	2DQ7	2	TK	EPHA8	3A7J	3
TK	FYN	2G2I	2	TK	EPHB4	2Z7S	3
TK	ABL1	2SRC	2	TK	EPHA7	1UNL	3
TK	SRC	3EYH	2	TKL	LIMK1	1YXT	3
TK	JAK1	3GVU	2				

Table S2. Top-ranked compounds in each group.

Compounds	Structure	Group	Rank in each group
ZINC02148815	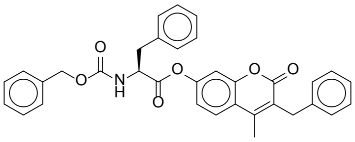	1	1
ZINC04833907	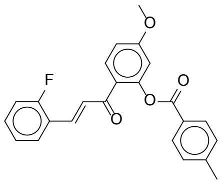	1	2
Rosmarinic acid	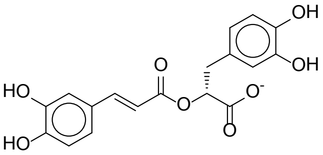	1	5
ZINC01875405	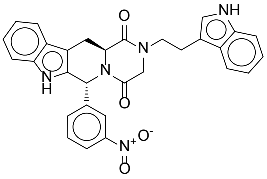	2	1
ZINC01830709	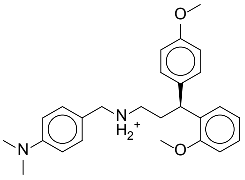	2	2
EGCG	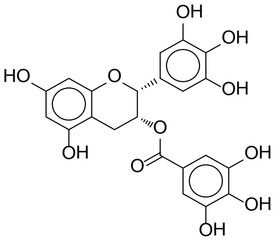	2	3
ZINC08792532	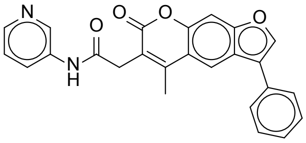	3	5
ZINC02030982	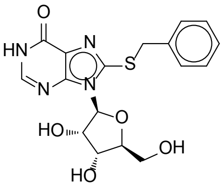	3	9
Quercetin	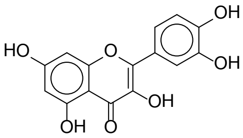	3	27

Table S3. Profiling results of the natural compounds against 64 protein kinases.

Family	Kinase	Remaining kinase activity (%)			Family	Kinase	Remaining kinase activity (%)		
		Quercetin	EGCG	Rosmarinic acid			Quercetin	EGCG	Rosmarinic acid
TK	EGFR	22[a]	≤0	9	CAMK	MKNK2	1	≥100	≥100
TK	INSR	21	25	≥100	CAMK	PIM1	1	≥100	≥100
TK	IGF1R	17	16	≥100	CAMK	PRKAA1	10	≥100	≥100
TK	ABL1	17	≥100	≥100	CK1	CSNK1G1	25	47	≥100
TK	ALK	34	≥100	≥100	CK2	VRK2	≥100	≥100	≥100
TK	BTK	35	≥100	≥100	CMGC	CDC2	14	≥100	≥100
TK	DDR2	≥100	≥100	≥100	CMGC	CDK5	9	≥100	≥100
TK	EPHA2	≥100	≥100	≥100	CMGC	CDK7	15	≥100	≥100
TK	FGFR1	6	≥100	≥100	CMGC	CLK3	6	44	≥100
TK	JAK3	≥100	≥100	≥100	CMGC	CSNK2A1	≤0	≥100	≥100
TK	KDR	3	≥100	≥100	CMGC	GSK3B	11	≥100	≥100
TK	KIT	28	4	≥100	CMGC	MAPK12	≥100	≥100	≥100
TK	LCK	17	≥100	≥100	CMGC	MAPK14	≥100	≥100	≥100
TK	MET	≥100	≥100	≥100	CMGC	MAPK3	34	13	≥100
TK	RET	7	≥100	≥100	CMGC	MAPK8	≥100	≥100	≥100
TK	SYK	20	17	20	Other	AURKA	11	≥100	≥100
TK	ZAP70	≥100	4	≥100	Other	CHUK	30	≥100	≥100
AGC	AKT1	≥100	≥100	≥100	Other	NEK2	≥100	≥100	≥100
AGC	AKT2	≥100	≥100	≥100	Other	NEK7	≥100	≥100	≥100
AGC	PDPK1	≥100	≥100	≥100	Other	PLK1	≥100	≥100	≥100
AGC	PRKCA	≥100	≥100	≥100	Other	WNK3	37	≥100	≥100
AGC	PRKCE	≥100	≥100	≥100	STE	MAP2K1	≥100	≥100	≥100
AGC	PRKCQ	≥100	≥100	≥100	STE	MAP2K6	≥100	≥100	≥100
AGC	ROCK1	≥100	≥100	≥100	STE	MAP3K5	≥100	≥100	≥100
AGC	RPS6KA1	4	≥100	≥100	STE	PAK4	≥100	≥100	≥100
CAMK	CAMK2G	3	≥100	≥100	STE	STK10	7	≥100	≥100
CAMK	CAMK4	28	≥100	≥100	STE	STK24	19	≥100	≥100
CAMK	CHEK1	31	≥100	≥100	TKL	LIMK1	12	≥100	≥100
CAMK	CHEK2	10	≥100	≥100	TKL	MAP3K7	44	≥100	≥100
CAMK	DAPK1	42	≥100	≥100	TKL	MAP3K9	18	≥100	≥100
CAMK	MAPKAPK2	≥100	≥100	≥100	TKL	RAF1	5	≥100	≥100
CAMK	MARK2	34	≥100	≥100	TKL	TGFBR1	≥100	≥100	≥100

[a] The percentage of remaining kinase activity when the compound concentration is 10 μM. A compound with ≤50% activity has an IC_{50} of ≤10 μM.

References

1. Madhusudan, Akamine P, Xuong NH, Taylor SS. Crystal structure of a transition state mimic of the catalytic subunit of cAMP-dependent protein kinase. *Nat. Struct. Biol.* **9**, 273-277 (2002).
2. Wheeler DL, Dunn EF, Harari PM. Understanding resistance to EGFR inhibitors-impact on future treatment strategies. *Nat. Rev. Clin. Oncol.* **7**, 493-507 (2010).
3. Russo MW, Lukas TJ, Cohen S, Staros JV. Identification of Residues in the Nucleotide Binding-Site of the Epidermal Growth-Factor Receptor Kinase. *J. Biol. Chem.* **260**, 5205-5208 (1985).
